# Machine learning identifies routine blood tests as accurate predictive measures of pollution-dependent poor cognitive function

**DOI:** 10.1038/s41598-026-64389-9

**Published:** 2026-07-29

**Authors:** Hamish Johnson, James Longden, Gary Cameron, Gordon D. Waiter, Fergal M. Waldron, Jenna M. Gregory, Holly Spence

**Affiliations:** 1 School of Geosciences, University of Aberdeen, Aberdeen, UK; 2 Theoretical Biophysics, Institute for Biology, Humboldt University of Berlin, Berlin, Germany; 3 Institute of Medical Sciences, School of Medicine, Medical Sciences and Nutrition, University of Aberdeen, Foresterhill, Aberdeen, AB25 2ZD UK; 4 Aberdeen Biomedical Imaging Centre, School of Medicine, Medical Sciences and Nutrition, University of Aberdeen, Aberdeen, UK

**Keywords:** Cognition, Dementia, Machine learning, Brain imaging, Atmospheric pollution, Social deprivation, Machine learning, Neuroscience, Cognitive ageing, Cognitive neuroscience

## Abstract

Several modifiable risk factors for dementias have been identified including socio-economic status and environmental exposures – however, how these population-level risks relate to individual risk remains elusive. To address this, we use random forest modelling to determine significant predictors of poor cognitive performance in on deeply phenotyped cohort of 324 individuals (age 61.6 ± 4.8 years; 150 males, 174 females) without extant neurological disease. 457 features were assessed including a comprehensive battery of imaging, blood, atmospheric pollutant and socio-economic measures. This approach where brain imaging, blood, socio-economic and environmental exposure measures were used to model cognitive performance was able to classify general cognition tertiles with 0.70 overall accuracy. Routinely assessed markers of anaemia including mean corpuscular haemoglobin were identified as predictors of poor general cognition and both extremes (low and high) of mean corpuscular haemoglobin concentration were associated with poor immediate recall (p = 0.0253). The predictors of poor cognition which consistently improved model performance across all models were measures of atmospheric pollution, in particular, lead, carbon monoxide, and particulate matter. Feature analysis demonstrated a significant negative relationship between low mean corpuscular haemoglobin concentration and high levels of atmospheric pollutants (p < 0.05) highlighting the potential of routinely assessed blood tests as predictive measures of pollution-dependent cognitive functioning, at an individual level. These data demonstrate how routine medical testing and local authority initiatives could identify at-risk individuals, highlighting the potential for targeted, cost-effective medical and social interventions to improve population cognitive health.

**Supplementary Information:**

The online version contains supplementary material available at https://doi.org/10.1038/s41598-026-64389-9.

## Introduction

Many risk factors for dementia have been identified including hypertension and diabetes, however there is often conflicting results across studies^1–4^. The Lancet commission report on dementia prevention highlighted twelve modifiable risk factors which are believed to account for 40% of dementias^3^. These included lower education, obesity, low social contact, and air pollution. By looking at modifiable factors that affect incident cognitive performance, we aim to define readily identifiable environmental and health factors responsible for poor cognitive performance to influence policy and improve cognitive health for those most at risk. To do this we assess a comprehensive battery of socio-demographic risk factors, brain imaging markers, blood markers and atmospheric pollutants in a deeply phenotyped cohort of older individuals.

Higher levels of social deprivation have previously been associated with many diseases including an increased risk of dementia^5,6^. In these studies, characterisation of socio-economic status varies widely from leaving education at an earlier age to social class, and there remains a lack of consensus regarding which specific socio-economic stressors may impact cognitive health most substantially. The current cohort allows the unique opportunity to combine comprehensive socio-demographic information in the form of the Scottish Index for Multiple Deprivation^7^ with other health indicators to identify areas where social change may benefit cognitive health. Whilst identification of specific social deprivation predictors of cognitive health is essential to determining policy and lifestyle change, it is important to also consider imaging or blood predictors that could be used as biomarkers of cognitive health. Brain imaging measures of regional volume and regional iron, as well as blood markers of anaemia and inflammation have previously been shown to have an association with dementia and so are also included in the current study^8–13^.

Exposure to air pollution has been associated with cognitive health variability with longitudinal studies showing that regardless of region of recruitment (i.e. area of dwelling), levels of particulate matter (PM2.5), carbon monoxide and nitrogen dioxide were associated with cognitive decline and dementia incidence^14^. Whilst several studies show a positive association between pollutant levels and rates of dementia, some studies showed a lack of association^15^ or a negative association^16^. These pollutants have been suggested to be detrimental to human health through activation pathways including oxidative stress and neuroinflammation induction^14,16^. This highlights the need for further study in a large deeply phenotyped cohort to elucidate the nuanced relationships between pollution and cognitive health.

**Fig. 1:**
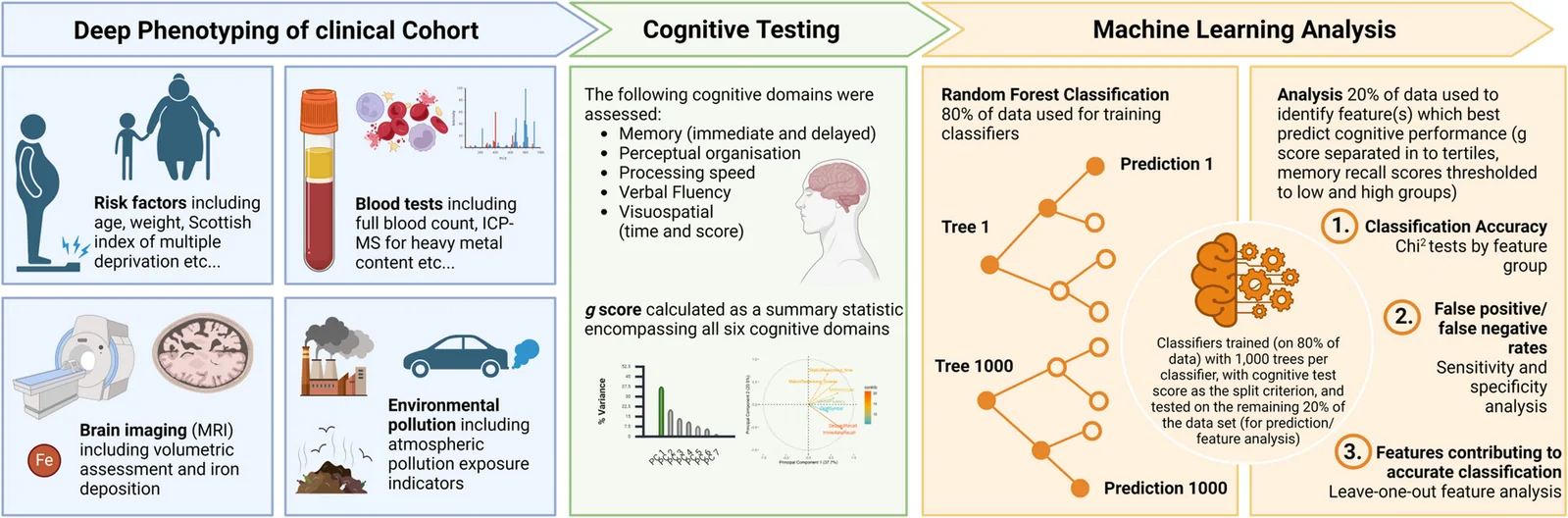
Cohort profiling and random forest modelling workflow.

Using a random forest approach which reduces investigator bias, here we assess a deeply phenotyped cohort of older individuals with no reported neurological disease to determine the strongest predictors of poor cognitive function to detect those at increased risk of dementia and other neurodegenerative disorders. Phenotyping of this cohort with brain imaging, blood sample testing, cognitive testing and spatially linked Scottish index of multiple deprivation and atmospheric pollution data allows for comprehensive modelling of cognitive predictors. Robust cognitive assessment comprised seven measures over five cognitive domains including memory and recall (immediate and delayed tests), perceptual organisation, processing speed, matrix reasoning (time and score) and verbal fluency. These seven measures were combined via principal component analysis to generate a *g* scorefor general cognition (*g*)^17,18^. Using this approach, we can gain insights into predictors of general cognitive health. We also analyse these cognitive domains separately where each domain represents a risk factor for dementia subtypes, for example, poorer memory score represents a higher risk for Alzheimer’s type dementia. We establish a random forest modelling approach to allow for non-biased feature identification, determining predictors of poor cognitive function. This study aims to establish risk factors for which routine clinical and local authority intervention could be taken to identify and protect ‘at risk’ populations (Fig. 1).

## Methods

### Cohort profile

Participants of the Aberdeen Children of the 1950’s (ACONF) study and their first-generation relatives who had given consent to be recontacted and still lived in Scotland with a community health index (CHI) number were recruited as part of the STratifying Resilience and Depression Longitudinally (STRADL) study^19^. Ethical approval was previously obtained from the Scotland A Research Ethics Committee (REC reference number 14/55/0039) and the local Research and Development offices. As outlined in the cohort profile^19^ informed consent was obtained from the participants (*n* = 1,188) for use of data in medical research into health, illness and medical treatment. Participants were informed their data would remain anonymous and would be added to those already held by Generation Scotland. All methods were performed in accordance with the relevant guidelines and regulations Participants were eligible for inclusion if they had MRI data suitable for quantitative susceptibility mapping (QSM) acquired at the Aberdeen study centre and did not report a neurological or neurodegenerative condition. Restriction to MRI data acquired at a single study centre was applied to ensure consistency of imaging acquisition procedures and scanner parameters across participants.A total of 324 individuals (61.6 ± 4.8 y/o; 150 males, 174 females) were included in this study and measurements were acquired between 2015 and 2017. A series of cognitive tests were performed on day of MRI and blood sampling. These included the Mill Hill Vocabulary test, the controlled word association task, the logical memory subset (immediate and delayed recall) and digit symbol coding task of the Wechsler adult intelligence scale III and the matrix reasoning test of the COGNITO psychometric examination^19^. Scottish Index of Multiple Deprivation (SIMD) was used as a relative measure of deprivation and was collected from the freely available database (www.simd.scot) based on the known postcode of the participants on the day of cognitive assessment, imaging and blood sampling. The 2016 SIMD measures were used as this was the closest available timepoint to MRI, blood and cognitive testing.

### Imaging

Imaging protocols are described fully by Habota et al., 2019. Briefly, images were acquired on a 3 T Philips Achieva TX-series MRI system (Philips Healthcare, Best, Netherlands) with a 32-channel phased- array head coil. 3D T1 weighted fast gradient echo images were acquired with the following parameters: 160 sagittal slices, TR = 8.2 ms, TE = 3.8 ms, TI = 1031 ms, FA = 8°, FOV = 240 mm, matrix size = 240 × 240, voxel size = 1.0 × 1.0 × 1.0 mm^3^, acquisition time = 5 min 38 s. MEGRE images were acquired with the following parameters: 130 axial slices, TR = 31, TE = 7.2/13.4/19.6/25.8 ms, FA = 17°, FOV = 230 mm, matrix size = 384 × 316, Voxel size = 0.3 × 0.3 × 1 mm^3^, acquisition time = 4 min 29s. Brain masks were generated using the brain extraction tool function of fsl software^20^. Phase images from MEGRE data were processed through a QSM pipeline, using the STISuite V3.0 QSM GUI pipeline with Laplacian based phase unwrapping, a VSHARP background field correction and the iLSQR QSM method to calculate susceptibility maps used to estimate iron content as described previously^18,21^.

### Plasma markers

Blood samples were taken from unfasted participants. A full blood count was carried out within NHS laboratories as described in the STRADL cohort profile^19^. C-reactive protein (CRP) levels were described here as undetectable (< 4 mg/L - below detection limit), normal (4–10 mg/L) or high (> 10 mg/L).

As described previously^12^, plasma samples were assessed for sTfR (RD194011100 - Oxford Biosystems Ltd UK), ferritin (RAB0197- 1KT, Merck Life Science UK Ltd) via enzyme linked immunosorbent assays (ELISA). Absorbance was read at 450 nm on a uQuant BioTek microplate spectrophotometer (Biotek Instruments, Inc). Macrophage colony-stimulating factor (M-CSF; Authentikine KE00184, Proteintech Group, Inc), IL1β (BMS224-2TEN – Thermo Fisher Scientific Inc.) and IL6 (BMS213-2TEN – Thermo Fisher Scientific Inc.) were also analysed via ELISA. Absorbance was read at 450 nm on an Infinite 200 PRO Tecan microplate reader.

Elemental analysis (Na, Mg, P, K, Ca, Mn, Fe, Co, Cu, Zn, As, Se, Mo, Cd, Hg and Pb) was carried out using an Agilent 7700x ICP-MS, following sample digestion with nitric acid and hydrogen peroxide.

### Environmental exposure estimation

Participant exposure to ambient atmospheric pollutants were estimated from modelled background air concentrations^22^ and atmospheric emission inventories^23^ available for the UK. Residential location at the time of assessment was used to link environmental exposure measures. As longitudinal residential histories were not available, these measures represent exposure at the time of assessment and may not reflect cumulative or lifetime exposure.

The modelled background air concentration maps provide estimates of ambient air pollutant (AAP) concentrations, incorporating both local point sources and distal sources when accounting for atmospheric dispersion^24^. Whereas the atmospheric emission inventory (AEI) maps represent the total mass of pollutant released to the atmosphere within a given grid cell from all sectors, and do not account for pollutant dispersion^25^. The spatially distributed time series data were both available at spatial resolutions of 1 × 1 km. Study participant residential addresses were mapped to the corresponding grid cell of each dataset. Here, the AAP dataset represents levels of pollutants in the air that people breath at their residential address whilst the AEI dataset represents potential exposure to pollutant emissions within ≤ 1 km. The annual limits are based on the UK The Air Quality Standards Regulations 2010 (Supplementary Table 1; Supplementary Fig. 1).

### Statistical analysis and machine learning

**Fig. 2:**
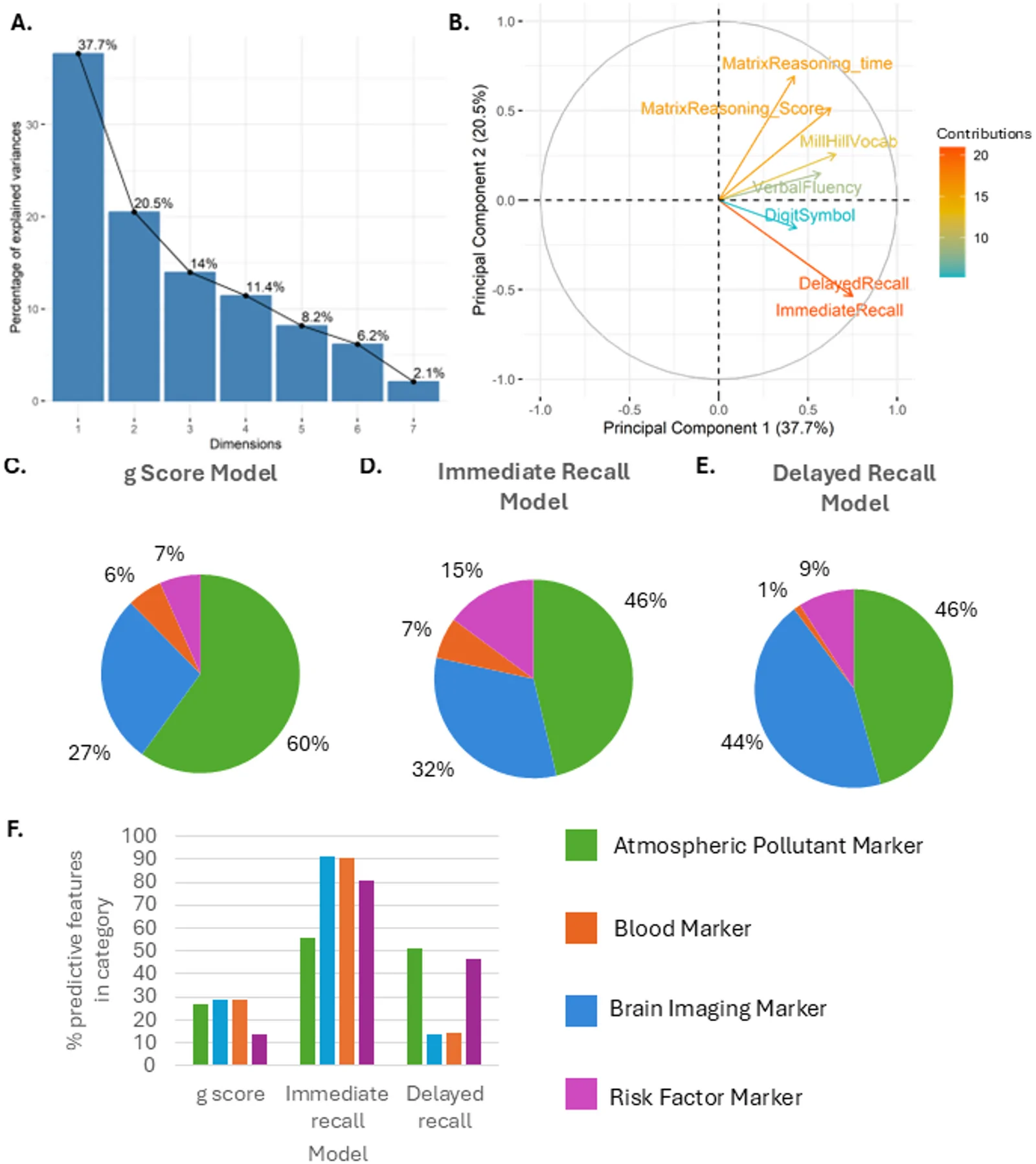
Pie charts to show percentage of predictors from each feature domain. Atmospheric pollutant markers constituted the largest domain of predictors in every model. (**A**) Scree plot to show the variance explained by each principal component. General cognition (*g* score) was defined as the first dimension (principal component 1). (**B**) Biplot to show the loadings of each included cognitive variable on the first principal component. (**C**) Percentage of predictors from each feature domain shown to predict *g* score for general cognition after leave-one-out analysis. (**D**) Percentage of predictors from each feature domain shown to predict immediate recall memory score after leave-one-out analysis. (**E**) Percentage of predictors from each feature domain shown to predict delayed recall memory score after leave-one-out analysis. (**F**) Percentage of predictive features within each category by model.

Principal component analysis was implemented in R statistical software^26^ with package ‘factoExtra’ and ‘Hmisc’ and visualised using ‘tidyverse’, ‘gridExtra’ and ‘corrplot’^27–31^ to generate a *g*score for general cognition from composite cognitive assessment scores (Mill Hill Vocabulary test, controlled word association task, logical memory subset (immediate and delayed recall), digit symbol coding task and the matrix reasoning test (score and time taken)^32^. Here, the *g* score was defined as the first principal component (Fig. 2A-B) generated following Z-standardisation and participants were divided into three classes based on tertiles of *g* score, where class one represented those within the highest tertile of *g* scores and class three represented those within the lowest tertile of *g* scores. Demographic information regarding each tertile is detailed in Table 1. For analysis of classifiers of individual cognitive assessment scores, the median integer score was used as a cut off for high and low groups.

**Table 1:** Cohort demographics in the low, medium and high *g* score tertiles respectively.

Age (mean ± sd)	Low	Medium	High
	62.1 ± 4.9	61.2 ± 3.8	61.4 ± 5.4
Sex (M: F)	59:49	44:64	47:61
Immediate Recall (mean ± sd)	12.6 ± 2.7	15.2 ± 2.4	18.7 ± 2.2
Delayed Recall (mean ± sd)	11.2 ± 2.9	14.2 ± 2.7	17.7 ± 2.2
Digit Symbol Task (mean ± sd)	60.6 ± 12.0	66.8 ± 12.6	72.0 ± 12.5
Matrix Reasoning (mean ± sd)	6.7 ± 2.1	8.75 ± 1.8	10.0 ± 2.1
Mill Hill Vocabulary Score (mean ± sd)	29.4 ± 3.4	31.9 ± 3.1	34.8 ± 3.2
Verbal Fluency (mean ± sd)	35.4 ± 9.5	42.4 ± 9.9	49.7 ± 11.0

The random forest approach was used to objectively evaluate all available features within the model, reducing the risk of introducing investigator bias whilst allowing identification of capturing complex, non-linear relationships and interactions. 457 features were extracted from clinical, imaging, plasma analysis and environmental exposure data and these data from 80% of the cohort were used to train random forest classifiers (Supplementary Table 2; Fig. 1). Missing values were handled internally by the random forest algorithm during split optimisation, rather than through prior imputation. No cognitive outcome variables contained missing values; therefore, all participants included in the analysis had complete cognitive assessment outcome data. Although the random forest algorithm can internally accommodate missing predictor data and the overall percentage missingness in this study was 0.64%, the possibility of missingness not at random (MNAR) cannot be excluded, and this may have influenced estimates of variable importance. Classification of subjects was performed using the random forest implementation in Knime 5.2.5^33^. Classifiers were trained with 1,000 trees per classifier and information gain as the split criterion and tested and validated on the remaining 20% of the data set. These parameters were used to ensure stable, convergent performance and ability to assess model validity in a cross-validation and an independent test set. Model performance was evaluated using sensitivity, specificity, and overall accuracy. These metrics were calculated on the held-out test set (20% of the cohort) following training on the remaining 80% of the data. In addition, cross-validation within the training set was performed to ensure model stability and to minimise overfitting (Tables 2, 4, 5, 6 and 3).

**Table 2:** Classification metrics of the random forest model for *g* score model cross-validation. Here, T1 is the tertile of the highest performing group and T3 is the tertile of the lowest performing group.

*n*	True Positives	False Positives	True Negatives	False Negatives	Precision	Sensitivity	Specificity	F-measure	Accuracy	Cohen’s kappa
T1	89	42	174	19	0.679	0.824	0.806	0.745		
T2	56	41	175	52	0.577	0.519	0.810	0.546		
T3	81	15	201	27	0.844	0.750	0.931	0.794		
									0.698	0.546

**Table 3:** Classification metrics of the random forest model for matrix reasoning task score cross-validation.

	True Positives	False Positives	True Negatives	False Negatives	Precision	Sensitivity	Specificity	F-measure	Accuracy	Cohen’s kappa
High	127	110	59	30	0.536	0.809	0.349	0.645		
Low	59	30	127	110	0.663	0.349	0.809	0.457		
									0.571	0.155

Finally, leave-one‐out analyses determined the change in model performance when each feature is excluded (Supplementary Tables 3–5). This approach allowed us to quantify the contribution of individual features to model performance and to identify those with the greatest impact on predictive accuracy. This analysis was performed for each cognitive assessment measure. Features that were shown as predictors in machine learning modelling with greatest magnitude improvement in model performance upon inclusion were carried forward for statistical comparison between cognitive groups. This analysis was visualised in R statistical software^26^ with the ggplot2 package^34^. Cognitive group comparison was completed for the features which demonstrated greatest magnitude of model improvement determined through leave-one-out analysis. Shapiro Wilk’s test was initially used to determine whether data distribution was parametric. Wilcox test or Pairwise Wilcox test with Holm post-hoc correction was used to analyse non-parametric data. *T*-test or ANOVA with Tukey-HSD post-hoc correction was used to analyse parametric data.

Measures related to mean corpuscular haemoglobin, together with pollution measures, were the only variables that consistently improved predictive performance across the models, relationships between these variables were explored further using linear and quadratic regression modelling employing the *lm* function in the R “stats” package^26^.

## Results

### Machine learning model classifies lowest cognition score group with high sensitivity and specificity

This non-biased machine learning approach where brain imaging, blood, socio-economic and environmental exposure measures were used to model cognitive performance was able to classify general cognition tertiles with 0.70 overall accuracy. The resultant model was most sensitive and specific in classifying those with the lowest cognitive scores (75.0% and 93.1% respectively; Table 2). When cognitive assessment measures were individually assessed, random forest models were sensitive and specific at classifying low immediate recall (68.3% and 82.5% respectively, Table 4) and delayed recall (69.5% and 80.1% respectively, Table 5) scores. However, modelling with the included 457 features lacked sensitivity when classifying low digit symbol task (41.2%, Table 6), matrix reasoning task (34.9%, Table 3), Mill Hill Vocabulary task (34.9%, Table 7) and controlled word association task (35.7%, Table 8) scores. Due to the reduced accuracy of the latter two models, feature analysis was not carried out for models of these cognitive test scores.

**Table 4:** Classification metrics of the random forest model for memory score (immediate recall) cross-validation.

	True Positives	False Positives	True Negatives	False Negatives	Precision	Sensitivity	Specificity	F-measure	Accuracy	Cohen’s kappa
High	113	60	129	24	0.653	0.825	0.683	0.729		
Low	129	24	113	60	0.843	0.683	0.825	0.754		
									0.742	0.490

**Table 5:** Classification metrics of the random forest model for memory score (delayed recall) cross-validation.

	True Positives	False Positives	True Negatives	False Negatives	Precision	Sensitivity	Specificity	F-measure	Accuracy	Cohen’s kappa
High	109	58	132	27	0.653	0.801	0.695	0.719		
Low	132	27	109	58	0.830	0.695	0.801	0.756		
									0.739	0.481

**Table 6:** Classification metrics of the random forest model for digit symbol task score cross-validation.

	True Positives	False Positives	True Negatives	False Negatives	Precision	Sensitivity	Specificity	F-measure	Accuracy	Cohen’s kappa
High	122	90	63	49	0.575	0.713	0.412	0.637		
Low	63	49	122	90	0.563	0.412	0.713	0.475		
									0.571	0.127

**Table 7:** Classification metrics of the random forest model for mill hill vocabulary task score cross-validation.

	True Positives	False Positives	True Negatives	False Negatives	Precision	Sensitivity	Specificity	F-measure	Accuracy	Cohen’s kappa
High	102	123	66	35	0.453	0.745	0.349	0.565		
Low	66	35	102	123	0.653	0.349	0.745	0.455		
									0.515	0.086

**Table 8:** Classification metrics of the random forest model for controlled word association task score cross-validation.

	True Positives	False Positives	True Negatives	False Negatives	Precision	Sensitivity	Specificity	F-measure	Accuracy	Cohen’s kappa
High	121	108	60	37	0.528	0.766	0.357	0.625		
Low	60	37	121	108	0.619	0.357	0.766	0.453		
									0.555	0.121

Brain imaging and atmospheric pollution markers represented the largest proportion of predictive markers (Fig. 2C-E) although it should be noted that these two categories also had the largest number of markers contributing to the model. When normalised by the number of markers in each category, around 27–29% of atmospheric pollution, brain imaging and blood markers were predictive of general cognition (*g* score), compared to only 13.5% of risk factor markers (Fig. 2F). More markers were predictive in general for immediate recall score with over 80% of brain imaging, blood and risk factor markers and over 50% of atmospheric pollution markers being predictive (Fig. 2F). Over 40% of atmospheric pollution markers and risk factor markers were predictive for delayed recall compared to less than 15% blood and risk factor markers (Fig. 2F).

### Specific measures of education and deprivation predicted immediate recall abilities more than other aspects of cognitive performance

Whilst only 7 out of 52 risk factor measures relating to socioeconomic and demographic features improved the model of general cognition score, 42 of these 52 risk factor measures improved the model of immediate recall score whilst 24 of these measures improved the delayed recall model (Fig. 2). Additionally, whilst in relation to g score classification, inclusion of risk factor measures individually improved model performance by < 7%, individual risk factor measures including SIMD zone improved the immediate recall model by up to 11.7% and individual employment measures improved the delayed recall model by 8%.

**Fig. 3:**
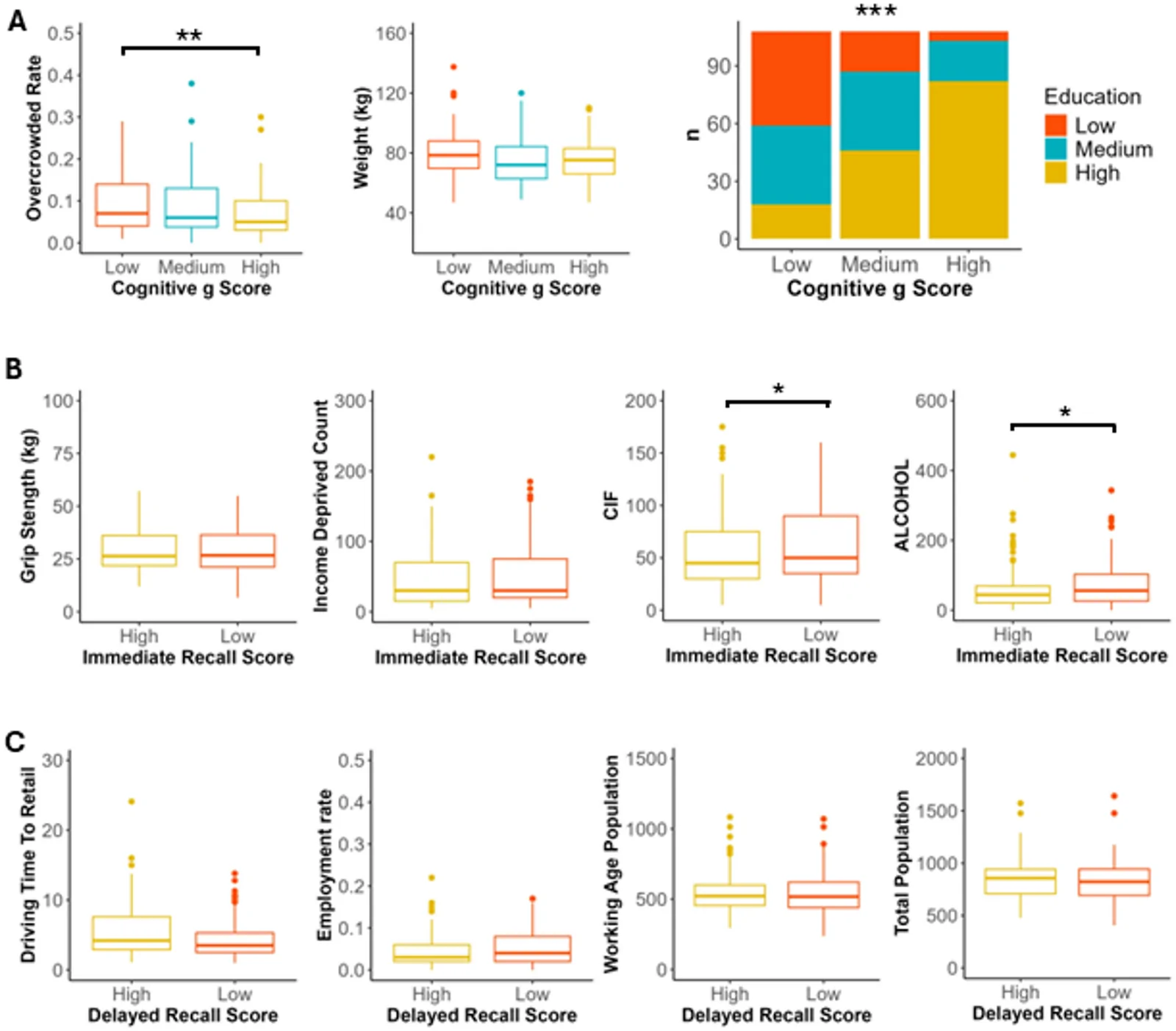
Specific measures of education and deprivation predicted immediate recall abilities more than other aspects of cognitive performance. (**A**) Three socio-economic factors markers that improved the random forest classifier for *g* score tertile group by the greatest amount in leave-one-out analysis. Box and whisker plots demonstrate that those with lower *g* score generally lived in regions with significantly higher rates of overcrowding (Pairwise Wilcoxon, Holm), non-significantly weighted more (Pairwise Wilcoxon, Holm) and had significantly lower levels of education (Chi Squared test). (**B**) Four socio-economic factors markers that improved the random forest classifier for immediate recall score group by the greatest amount in leave-one-out analysis. Box and whisker plots demonstrate that those with lower immediate recall score had non-significantly lower grip strength and lived in regions with significantly higher CIF (comparative illness factor; age and sex-standardized ratio of individuals with work-limiting morbidity and disabilities) and hospital stays related to ALCOHOL (prevalence of hazardous or harmful drinking) and non-significantly higher income deprivation (Pairwise Wilcoxon, Holm). (**C**) Four socio-economic factors markers that improved the random forest classifier for immediate recall score group by the greatest amount in leave-one-out analysis. Feature comparison demonstrated no significant difference between employment rate, working age or total population between delayed recall score groups or between driving time to retail in high compared to low delayed recall score groups once outlier was removed. (Pairwise Wilcoxon, Holm). *p < 0.05, **p < 0.01, ***p < 0.001.

Measures of school attainment, rate of overcrowding and number of homes with non-central heating in the participants SIMD region improved model performance for classifying poor general cognition (Supplementary Table 3). Age, sex and education also improved general cognition model performance, however, other socioeconomic and demographic factors included in the model for classifying general cognition did not improve model performance. After post-hoc analysis there was a significantly lower education level in those with low general cognition compared to those with high general cognition (*χ*^*2*^ = 89.748, *p* < 0.001; Fig. 3A). Those with low *g* score also lived in regions with significantly higher rate of overcrowding compared to those with high *g* scores (*p* = 0.007; Fig. 3A). There was no significant difference between the weight of participants dependent on general cognition score tertile (Fig. 3A).

In the immediate recall model, grip strength, income deprivation, comparative illness factor and emergency hospital visits resulting from alcohol were the strongest predictors. Feature comparison exhibited significantly higher comparative illness factor (age and sex-standardized ratio of individuals with work-limiting morbidity and disabilities) and number of emergency hospital stays due to alcohol in the low compared to high immediate recall score group (*p* = 0.046 and *p* = 0.023 respectively; Fig. 3B). There was no significant difference between grip strength or income deprivation between low and high immediate recall performers.

The risk factor predictors for delayed recall score which improved the model by the greatest magnitude were driving time to retail, employment rate, working age population and total population. Feature comparison demonstrated no significant difference between employment rate, working age or total population between delayed recall score groups or between driving time to retail in high compared to low delayed recall score groups once outlier was removed.

### Atrophy, swelling and iron content of specific brain regions predicted poorer cognitive performance

Whilst 29 out of 116 imaging measures improved the model of general cognition score, 91 of the 116 imaging improved the model of immediate recall score whilst 16 of these measures improved the delayed recall model (Fig. 2). Imaging measures were important features in the immediate recall classification model, with cortical volume measures individually improving model performance by 17.65%. Whilst having a lesser magnitude of effect on the g score and delayed recall classification models, inclusion of amygdala and thalamus iron measures improved delayed recall model performance by 8%, whilst cortical volume measures improved the g score model performance by 6.08%.

**Fig. 4:**
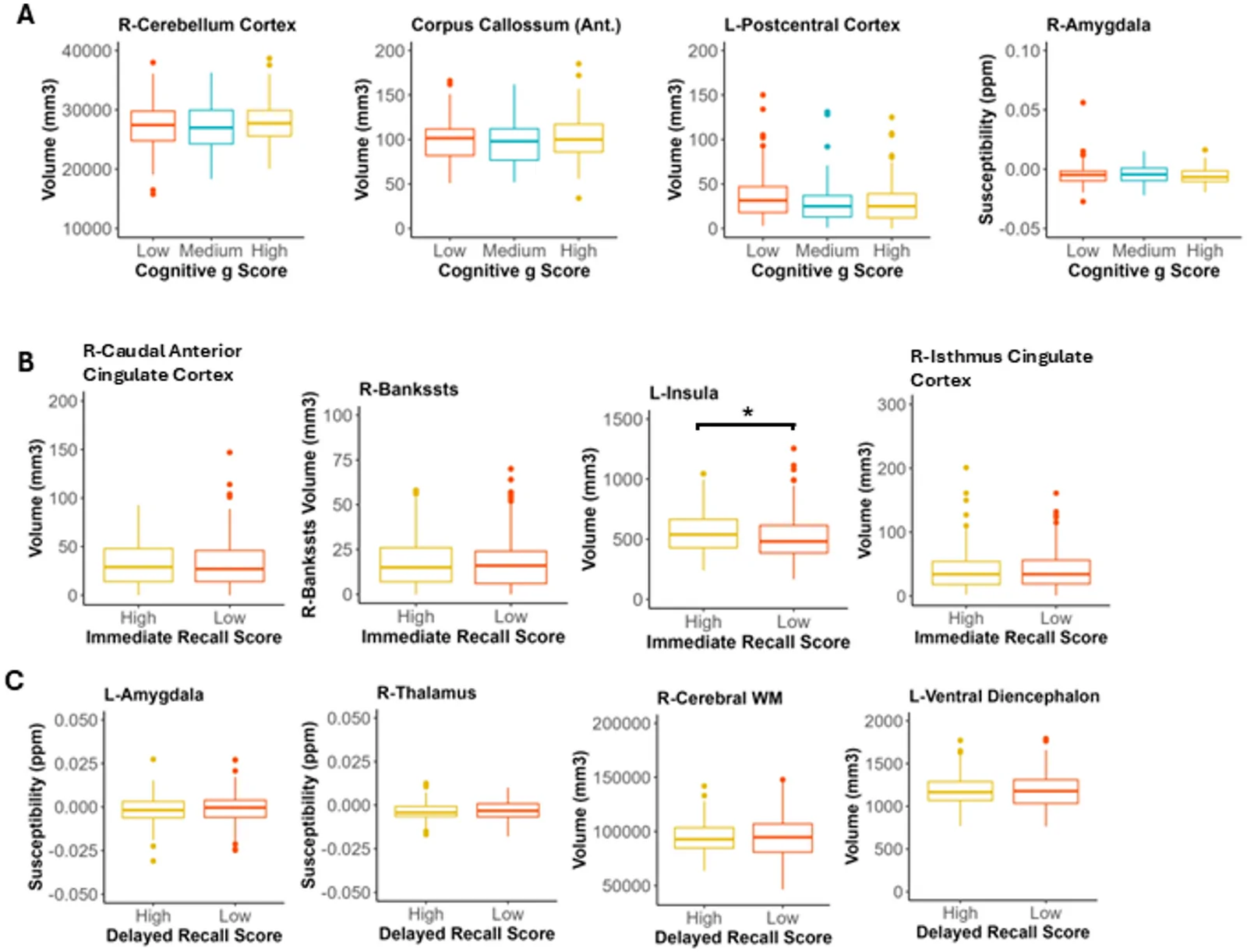
Atrophy, swelling and iron content of specific brain regions predicted poorer cognitive performance. (**A**) Four brain imaging measures that improved the random forest classifier for *g* score tertile group by the greatest amount in leave-one-out analysis. Box and whisker plots demonstrate that those with lower *g* score generally had non-significantly lower volumes of large regions such as the right cerebellar cortex and anterior corpus callossum, but non-significantly higher volume of the left postcentral cortex. Those with lower *g* scores also had non-significantly higher levels of iron in the right amygdala (Pairwise Wilcoxon, Holm). (**B**) Four brain imaging measures that improved the random forest classifier for immediate recall score group by the greatest amount in leave-one-out analysis. Box and whisker plots demonstrate that those with lower immediate recall score non-significantly higher volumes of the right caudal anterior cingulate cortex but significantly lower insula volume and non-significantly lower volume of the bankssts cortex and right isthmus cingulate cortex (Pairwise Wilcoxon, Holm). (**C**) Four brain imaging measures that improved the random forest classifier for immediate recall score group by the greatest amount in leave-one-out analysis. Box and whisker plots demonstrate that those with lower delayed recall score exhibit non-significantly higher levels of iron in the left amygdala (Pairwise Wilcoxon, Holm) and right thalamus (ANOVA, Tukey-HSD) and non-significantly lower volumes of the left ventral diencephalon (Pairwise Wilcoxon, Holm) and right cerebral white matter (ANOVA, Tukey-HSD). *p < 0.05, **p < 0.01, ***p < 0.001.

Volumes of the right cerebellum (cortex), corpus callossum and left postcentral cortex and iron in the right amygdala improved model classification performance for poor general cognition (Supplementary Table 3; Fig. 4A). There were no significant differences in these imaging measures between *g* score groups. In the immediate recall model, right caudal anterior cingulate cortex, right Bankssts cortex, left insula and right isthmus cingulate cortex volumes were the strongest predictors. Feature comparison exhibited significantly lower left insula cortex in the low compared to high immediate recall score group (*p* = 0.025; Supplementary Table 4; Fig. 4B). There was no significant difference between other volumes in low compared to high immediate recall performers. The strongest imaging predictors for delayed recall score were iron in the left amygdala and right thalamus and volume in the right cerebral white matter and left ventral diencephalon. Feature comparison demonstrated no significant differences between these measures in the low delayed recall group compared to the high delayed recall group (Supplementary Table 5; Fig. 4C).

### Blood measures of anaemia and chronic inflammation consistently improved model performance in predicting poorer cognitive performance across all three cognitive domains

Out of the 21 blood markers assessed, 6 improved the model of general cognition score, 19 improved the model of immediate recall score whilst only 3 improved the delayed recall model. Measures of mean corpuscular haemoglobin and CRP were predictors across each of the three models (Fig. 2). In fact, mean corpuscular volume contributed the greatest magnitude improvement of all features in the g score model (12.08%).

**Fig. 5:**
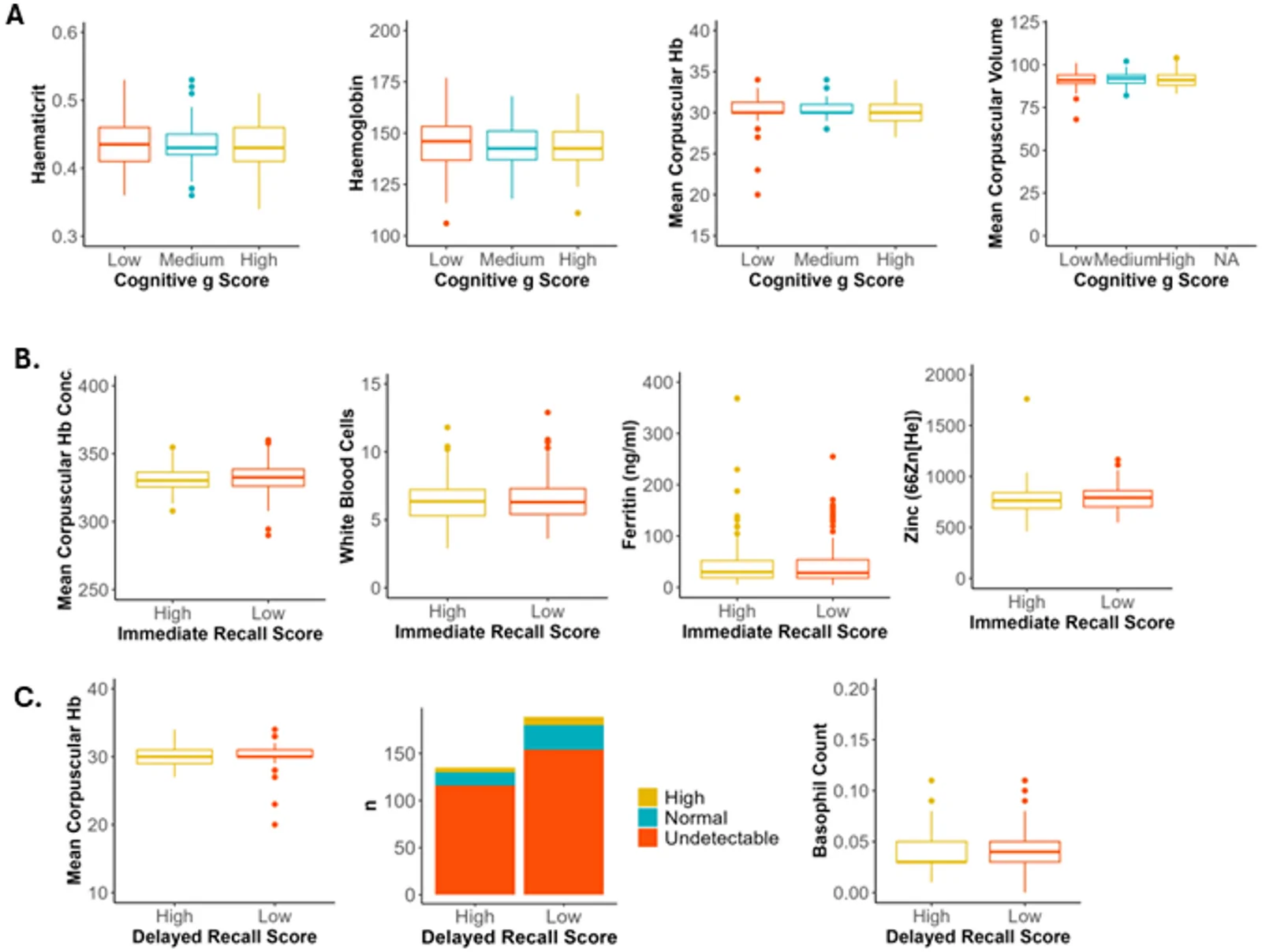
Blood measures of anaemia and chronic inflammation consistently improved model performance in predicting poorer cognitive performance. (**A**) Four blood markers that improved the random forest classifier for *g* score tertile group by the greatest amount in leave-one-out analysis. Box and whisker plots demonstrate that those with lower *g* score had non-significantly higher haemoglobin, haematocrit and mean corpuscular haemoglobin, but non-significantly lower mean corpuscular volume (Pairwise Wilcoxon, Holm). (**B**) Four blood markers that improved the random forest classifier for immediate recall performance by the greatest amount in leave-one-out analysis. Box and whisker plots demonstrate that those with lower immediate recall score had non-significantly higher mean corpuscular haemoglobin concentration, white blood cell count and plasma Zinc but non-significantly lower plasma ferritin (Pairwise Wilcoxon, Holm). (**C**) Three blood markers that improved the random forest classifier for delayed recall performance in leave-one-out analysis. Box and whisker plots demonstrate that those with lower delayed recall score had non-significantly higher mean corpuscular haemoglobin, basophil count (Pairwise Wilcoxon, Holm) and C-reactive protein (Chi squared test). *p < 0.05, **p < 0.01, ***p < 0.001.

Measures of haematocrit, haemoglobin, mean corpuscular haemoglobin and mean corpuscular volume each improved model performance for classifying poor general cognition (Supplementary Table 3; Fig. 5A). In the immediate recall model, mean corpuscular haemoglobin, white blood cell count, ferritin and zinc levels were the strongest predictors (Supplementary Table 4; Fig. 5B). The strongest blood marker predictors for delayed recall score were mean corpuscular haemoglobin, CRP and basophil count (Supplementary Table 5; Fig. 5C). Feature comparison demonstrated no significant differences between blood markers by *g* score, immediate recall or delayed recall group.

### Environmental exposure to heavy metals, PM25, SO_2_ and CO were predictors of poor cognitive performance across all models which improved model performance by the greatest magnitude

Across all three assessed models, atmospheric pollutant exposure consistently improved model performance by the greatest magnitude across all three considered models. Of 254 measures of atmospheric pollutant levels in the region of dwelling, 63 measures improved the model of general cognition score, 131 measures improved the model of immediate recall score whilst 120 measures improved the delayed recall model (Fig. 2). Upon feature analysis, measures of Arsenic, cadmium, copper, lead, mercury and PM2.5 were consistently identified as predictors of poorer *g* score, immediate recall score and delayed recall score demonstrating up to 9.08%, 9.80% and 14% improvement to each model respectively upon inclusion of individual measures.

**Fig. 6:**
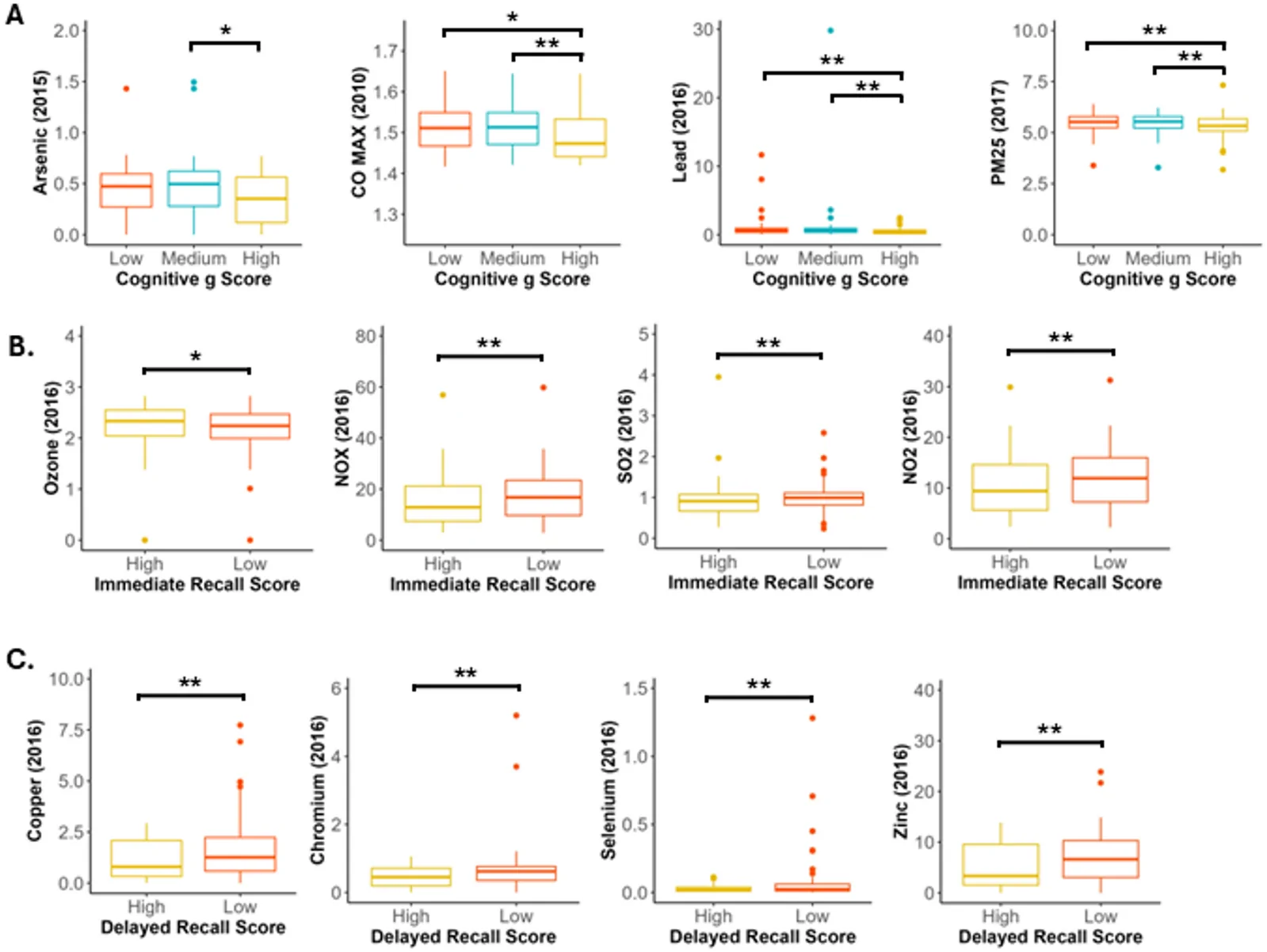
Environmental exposure to heavy metals, PM25, SO2 and CO were consistently the predictors of poor cognitive performance which improved model performance by the greatest magnitude. (**A**) Four modelled air quality and emissions measures that improved the random forest classifier for *g* score group by the greatest amount in leave-one-out analysis. Box and whisker plots demonstrate that those with lower *g* score lived in regions with significantly higher levels of Arsenic, carbon monoxide, lead and particulate matter in the year of measurement closest to the year of cognitive assessment (Pairwise Wilcoxon, Holm). (**B**) Four modelled air quality and emissions measures that improved the random forest classifier for immediate recall score by the greatest amount in leave-one-out analysis. Box and whisker plots demonstrate that those with lower immediate recall score lived in regions with significantly higher levels of NOX, NO2 and SO2 but significantly lower levels of Ozone in the year of measurement closest to the year of cognitive assessment (Pairwise Wilcoxon, Holm). (**C**) Four modelled air quality and emissions measures that improved the random forest classifier for immediate recall score by the greatest amount in leave-one-out analysis. Box and whisker plots demonstrate that those with lower delayed recall score lived in regions with significantly higher levels of copper, chromium, selenium and zinc in the year of measurement closest to the year of cognitive assessment (Pairwise Wilcoxon, Holm). *p < 0.05, **p < 0.01, ***p < 0.001.

Levels of arsenic, carbon monoxide, lead and PM2.5 in the years of measurement closer to those of cognitive testing improved model classification performance for poor general cognition. Significantly lower levels of arsenic (2015), carbon monoxide (2010), lead (2016) and PM2.5 (2017) were observed in the high *g* score group compared to the medium *g* score group (*p* = 0.013, *p* = 0.005, *p* = 0.003, *p* = 0.008 respectively; Fig. 6A). Significantly higher levels of carbon monoxide (2010), lead (2016) and PM2.5 (2017) were observed in the low *g* score group compared to the high *g* score group (*p* = 0.011, *p* = 0.003, *p* = 0.009 respectively; Supplementary Table 3; Fig. 6A).

In the immediate recall model, ozone, oxides of nitrogen, nitrogen dioxide and sulphur dioxide were the strongest predictors. Feature comparison revealed significantly lower levels of ozone in the low compared to high immediate recall group (*p* = 0.043; Fig. 6B). There were significantly higher levels of NOX, NO2 and SO2 in the low immediate recall score group compared to the high immediate recall score group (*p* = 0.008, *p* = 0.008, *p* = 0.006 respectively; Supplementary Table 4; Fig. 6B).

The strongest imaging predictors for delayed recall score were copper, chromium, selenium and zinc. Feature comparison demonstrated higher levels of copper, chromium, selenium and zinc in the low delayed recall group compared to the high delayed recall group (*p* = 0.001, *p* = 0.008, *p* = 0.006, *p* = 0.001 respectively; Supplementary Table 5; Fig. 6C).

### Low mean corpuscular haemoglobin concentration is a biomarker of pollution exposure and poor cognition

**Fig. 7:**
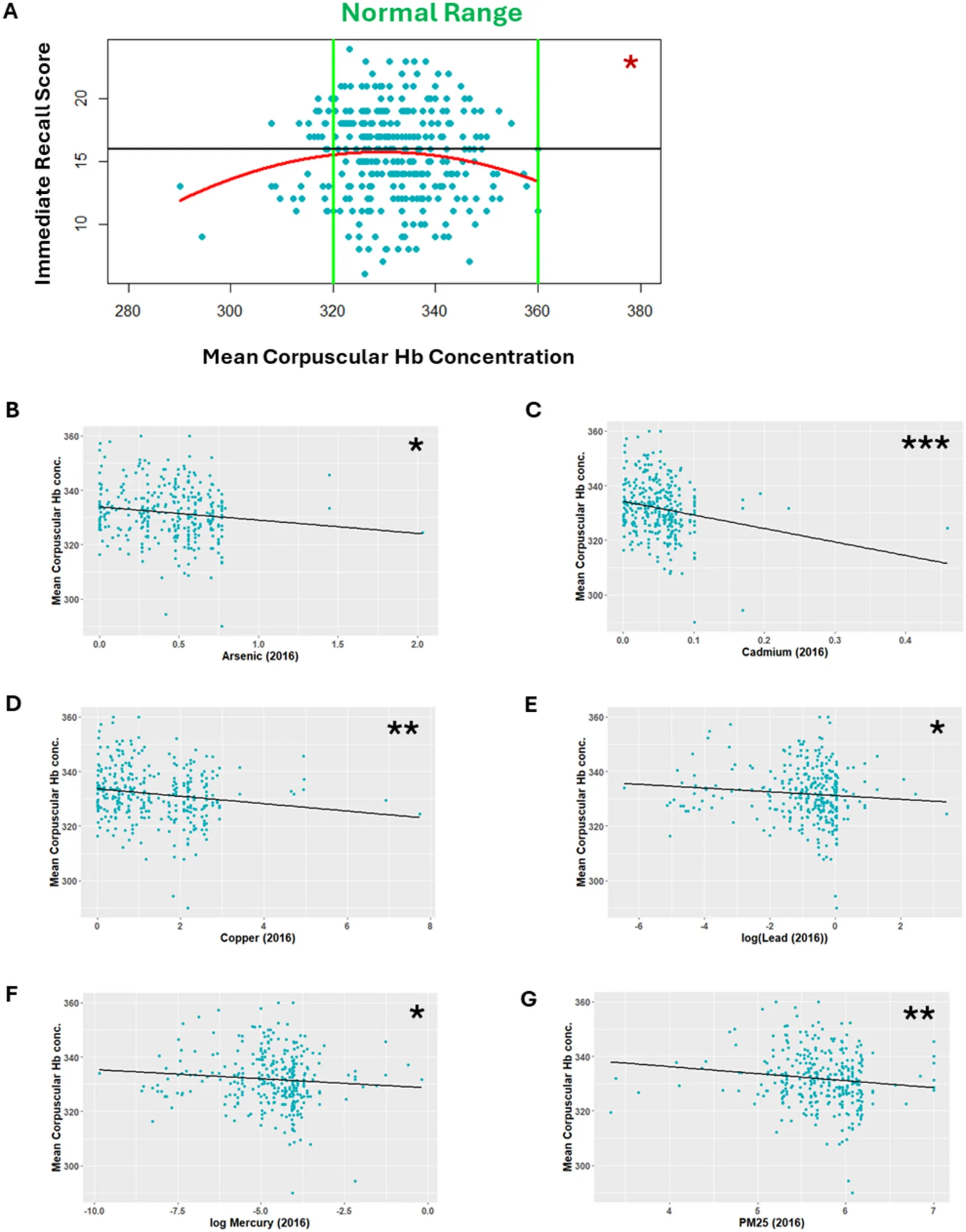
Clinically low mean corpuscular haemoglobin concentration is associated with lower immediate recall score and increased levels of predictive pollutants. (**A**) Mean corpuscular haemoglobin demonstrates a quadratic relationship with immediate recall score (*p* = 0.0253). In the year of measurement that was predictive closest to the years of cognitive assessment lower mean corpuscular haemoglobin was significantly associated with increased dwelling region atmospheric levels of (**B**) Arsenic (*p* = 0.018, *ρ* = −0.132), (**C**) Cadmium (*p* < 0.001, *ρ* = −0.200), (**D**) Copper (*p* = 0.006, *ρ* = −0.154), (**E**) Lead (log transformed, *p* = 0.019, *ρ* = −0.131), (**F**) Mercury (log transformed, *p* = 0.034, *ρ* = −0.118), (**G**) PM25 (*p* = 0.003, *ρ* = −0.164). *p < 0.05, **p < 0.01, ***p < 0.001.

As mean corpuscular haemoglobin measures and levels of arsenic, cadmium, copper, lead, mercury and PM25 in the region of dwelling were key predictive features across all three models of cognitive health, the relationships between these markers and cognitive assessment measures were assessed. Whilst MCHC and MCH were not significantly associated with general cognition score, there was a significant quadratic relationship between MCHC and immediate recall memory score (Fig. 7A; Supplementary Fig. 2). Cook’s distance was used to assess influence of extreme points and although some observations lie at the extremes of the observed MCHC range, the reported associations were not driven by the visually distinct observations. Low MCHC was also significantly correlated with increased exposure with increased dwelling region atmospheric levels of arsenic (*ρ* = −0.132, *p* = 0.018; Fig. 7B), cadmium (*ρ* = −0.200, *p* < 0.001; Fig. 7C), copper (*ρ* = −0.154, *p* = 0.006; Fig. 7D), lead (log transformed, *ρ* = −0.131, *p* = 0.019; Fig. 7E), mercury (log transformed, *ρ* = −0.118, *p* = 0.034; Fig. 7F) and PM25 (*ρ* = −0.164, *p* = 0.003; Fig. 7G). It should be noted that these significant correlations appear to be largely driven by those living in areas of extremely high pollution exposure. Our results show that in areas with higher levels of atmospheric pollution individuals had generally lower MCHC (Fig. 7) and generally lower memory score (Fig. 6). MCHC could therefore represent a biomarker for pollution-related poor memory function.

## Discussion

Here, we use a non-biased machine learning approach to identify key predictors of cognitive performance in a unique birth cohort with detailed demographic information. We show differences in predictive measures for different cognitive domains, where the highest number of predictors were identified for immediate recall scores. This suggests that different sets of variables are associated with performance across cognitive domains. In particular, measures related to combustion emissions and anaemia were key predictors of general cognitive performance, specific imaging markers were most predictive of immediate recall, and atmospheric pollutants associated with smelting and pesticide use were most predictive of delayed recall. Importantly, we observed that lower mean corpuscular haemoglobin levels were associated with higher measures of atmospheric arsenic, cadmium, copper, lead, mercury and PM2.5 in the region of dwelling, which were also associated with poorer memory performance. These findings suggest that mean corpuscular haemoglobin concentration may represent a candidate, routinely assessed marker associated with exposure-related differences in cognitive performance in populations living in areas of higher pollution.

### Poor geographic, health, education and income access are associated with poorer cognitive performance

Measures indicative of geographic access, health and income were specifically predictive of memory scores. This suggests a heavier impact of social deprivation on memory aspects of cognition. In the general cognition model, only 3 measures of social deprivation were predictive of poorer *g* score, however, 41 social-deprivation measures were predictive of specific memory scores. As immediate recall tasks have shown greatest sensitivity of predicting dementia^35^, this result indicates that social deprivation is a key risk factor for dementia. The three measures consistently predictive of performance across models (school attainment, overcrowding and number of homes with non-central heating) highlight that education and poverty indicators likely play a key role in determining general cognitive health.

### Atrophy and swelling detected on brain MRI are associated with poor cognitive function

There were also differences in the imaging markers predictive of general cognition compared to specific measures of memory performance. Whilst there were consistencies across models in the links between the volumes of regions involved in connectivity and signal relay such as the corpus callossum and thalamus, there were some regions such as the temporal cortex and inferior parietal cortex which were only predictive in classifying immediate recall memory score, regions that have been previously shown to play specific roles in memory function^36,37^. Atrophy of large regions such as the cerebellum was shown to predict general cognitive performance as well as specific memory scores. As these large regions can be visualised at a lower resolution, this opens opportunities for monitoring with low field MRI which is less expensive and more portable than conventional 3 T and above MRI, with implications for improving health equity.

Further, whilst atrophy of most regions was associated with poorer cognitive performance, consistent with previous studies^38–40^, some regions such as the inferior temporal cortex demonstrated association between increased volume and poorer cognitive performance. Importantly, this suggests that swelling, rather than atrophy, of some regions may be associated with poorer cognitive performance. This local swelling could be caused by inflammation of the myelin sheath which has been shown to occur during traumatic brain injury leading to local swelling effects and accumulation of proteins^41^.

### Living in regions with high vehicle emissions, refuse incineration and fossil fuel combustion may increase risk of poor cognitive health

Increased measures of atmospheric arsenic, cadmium, copper, lead, mercury and PM2.5 in the region of dwelling are associated with poorer cognitive performance. Exposure to air pollutants, including PM2.5 and associated heavy metals such as arsenic, cadmium, lead, mercury and copper, has been associated with impaired cognitive function and increased risk of neurodegenerative disease, with proposed mechanisms including oxidative stress and neuroinflammation^14,16,52^. Here, these association holds for measurement of pollutants not only in the years closest to cognitive testing but also those measured years before cognitive testing. This is consistent with previous studies that have demonstrated a 4–10-year lag in the association between PM2.5 and memory decline^15,42^. As demonstrated by Jung et al. (2015), there may be a threshold of certain pollutants that must be met before an effect on cognitive health can be observed^43^. This may explain why pollutants were not predictive of cognitive decline in some years. It is important to not that the inclusion of environmental exposure measures from multiple time windows (including 2018–2020) was intended to ssess whether pollution estimates derived from different periods within the same geographic region would show consistent associations with cognitive performance, and whether model performance was sensitive to the temporal window used for exposure assignment. These results suggest that pollutant exposure estimates may capture longer-term spatial patterns rather than time-specific levels, with model performance remaining robust across different exposure windows.

Arsenic, cadmium, lead and mercury have previously been identified as significant to public health and increased atmospheric levels of these four specific heavy metals in the UK has been linked with a rise in refuse incineration, steel production, fossil fuel combustion^44,45^. Some studies suggest that a combination of arsenic, cadmium and lead may increase neurotoxicity through combined alteration of the ERK-JNK pathway, promoting apoptosis^46^. It may be therefore, that the threshold for each of these exposures is lower than other heavy metals. Due to the charged nature of heavy metals including copper, they are able to strongly bind to particulate matter and are in turn known to increase reactive oxygen species and oxidative stress which may explain their associations with cognitive performance observed in this study^47^.

### Routinely assessed measures of anaemia represent an accurate predictive measure of pollution-dependent poor cognitive function

We show that mean corpuscular haemoglobin measures consistently improved model performance in predicting poorer cognitive health across all three assessed models and after feature analysis it was determined that MCHC demonstrated a significant quadratic relationship with immediate recall memory score. This finding corroborates previous studies that show both low and high levels of haemoglobin as associated with dementia in a u-shaped association, whilst anaemia was associated with increased risk of dementia^11,48^ and highlights the potential for identifying at risk groups through routine in clinic blood testing. It is thought that higher MHCH is indicative of microcytic anaemia and/or polycythaemia often caused by iron deficiency or excessive alcohol consumption/smoking respectively, are detrimental to cognitive health likely due to impaired oxygen carrying capacity^49^. However, low MCHC is associated with iron-deficiency anaemia and we show that lower MCHC is significantly associated with increased levels of dwelling region atmospheric pollutant levels, particularly in those with extremely high pollutant levels. This fits with previous findings that suggest PM25 and cadmium exposure in particular can induce iron-deficiency anaemia^50–53^. Our results highlight the potential for MCHC as a routinely assessed biomarker of pollution exposure related cognitive risk.

Whilst heavy metal exposure (e.g. atmospheric lead) was a strong predictor of poor cognitive performance, blood levels of such heavy metals were not predictive of general cognition. It is likely that heavy metal exposure results in end organ accumulation of these heavy metals and so blood levels were not reflective of total body levels^54^. However, blood markers of heavy metals were predictive of memory scores, suggesting that this may be easier to track in those at risk of Alzheimer’s type dementia. Atmospheric pollutant measures could be utilised to identify at risk groups and “green corridors” of low levels of predictive pollutants to inform public footpaths and walk-to-school or -work routes. Recent studies have shown promise in combatting the effects of exposure to pollutants such as arsenic^55^. Therefore, if at risk groups are identified through atmospheric pollutant mapping, there is opportunity for targeted preventative treatment strategies.

It is important to consider the context of the studied cohort when interpreting these results. Whilst this cohort offered a fairly homogeneous foundation population for determining exposure influence, there exists an inequality in the dementia incidence across racial and ethnic groups which could not be assessed due to the lack of diversity within this regional birth cohort^56^. Another limitation of the present study is that analyses were restricted to participants with MRI data suitable for QSM analysis acquired at a single study centre. Consequently, potential selection bias cannot be excluded, as participants who underwent MRI and had usable imaging data may differ systematically from those who did not, potentially limiting generalisability. It should additionally be taken into account that non-neurological diseases such as Diabetes mellitus may confound an individual’s risk for dementia, however, such disease status was not included in this study due to the lack of data on non-neurological disease status in this cohort^57^. Additionally, environmental exposure estimates were based on residential location at the time of assessment and therefore reflect contemporaneous rather than lifetime exposure. As longitudinal residential histories were not available, this may introduce exposure misclassification where participants may have relocated or experienced varying environmental conditions over time. However, given the relatively stable geographic distribution of the cohort, this approach provides a reasonable proxy for environmental exposure at the time of assessment. Furthermore, given the large number of clinical, environmental and biomarker variables included, there is potential for collinearity between predictors. While the random forest approach is robust to multicollinearity in terms of predictive performance, correlated features may influence the stability and interpretability of feature importance measures. This may also explain the observation that several features demonstrated similar magnitudes of model improvement, reflecting shared variance among correlated predictors rather than independent effects and this should be considered when interpreting the contribution of individual predictors.

## Conclusions

This study highlights the potential relevance of modifiable risk factors in relation to cognitive performance, which may inform future prevention strategies and economically accessible clinical approaches. Across models for general cognitive performance, immediate and delayed recall, levels of mean corpuscular haemoglobin and atmospheric pollutants including PM2.5, arsenic and lead in the year of measurement and in years prior to measurement were the strongest predictors of cognitive performance with greatest magnitude contribution of model performance. These findings indicate that increased exposure to these pollutants is associated with poorer cognitive outcomes, and that routinely measured mean corpuscular haemoglobin concentration may serve as a candidate marker of exposure-related risk. Further longitudinal study is vital to understand the mechanisms of these factors in affecting brain health, as well as to determine how such information may be used to identify and protect at-risk populations on a local, national and international scale.

## Supplementary Information

Below is the link to the electronic supplementary material.

Supplementary Material 1

## Data Availability

The data collected in the STRADL study have been incorporated in the larger Generation Scotland dataset. Non-identifiable information from the Generation Scotland cohort is available to researchers in the United Kingdom and to international collaborators through application to the Generation Scotland Access Committee (access@generationscotland.org). Generation Scotland operates a managed data access process including an online application form, and proposals are reviewed by the Generation Scotland Access Committee.

## Code availability

Code has been deposited in https://github.com/HollySneuro/Johnson2026; DOI: https://doi.org/10.5281/zenodo.19188790.
