# Supplementary material for: Machine learning identifies routine blood tests as accurate predictive measures of pollution-dependent poor cognitive function
Source: Sci Rep. 2026 Jul 29;16:34935. doi: 10.1038/s41598-026-64389-9 (PMC13713566; doi:10.1038/s41598-026-64389-9)
Supplement: Supplementary file 1 [file 41598_2026_64389_MOESM1_ESM.pdf]

Supplementary Table 1. Annual limits for ambient atmospheric pollutant measures. [a] Total days of exposure where 8-hour mean concentrations are ≥120 μg m^{-3}

	Variable	Metric	Units	Start	End	Annual limit
DEFRA modelled background atmospheric pollution data	Benzene	Annual mean	μg m^{-3}	2003	2022	5 μg m^{-3}
	Carbon monoxide	Annual mean	mg m^{-3}	2002	2010	10 mg m^{-3}
		Maximum 8-hour mean	mg m^{-3}	2002	2010	-
	NO_2	Annual mean	μg m^{-3}	2001	2022	40 μg m^{-3}
	NO_X	Annual mean	μg m^{-3}	2001	2022	40 μg m^{-3}
	Ozone	Total[a]	Days	2003	2022	25 days
	$PM_{2.5}$	Annual mean	μg m^{-3}	2002	2022	25 μg m^{-3}
	SO_2	Annual mean	μg m^{-3}	2002	2022	125 μg m^{-3}
NAEI total atmospheric pollutants emissions	Arsenic	Total	kg/km^2	2010	2020	6 μg m^{-3}
	Cadmium	Total	kg/km^2	2010	2020	5 μg m^{-3}
	Chromium	Total	kg/km^2	2010	2020	-
	Copper	Total	kg/km^2	2010	2020	-
	Lead	Total	kg/km^2	2010	2020	0.5 μg m^{-3}
	Mercury	Total	kg/km^2	2010	2020	-
	Nickel	Total	kg/km^2	2010	2020	20 μg m^{-3}
	Selenium	Total	kg/km^2	2010	2020	-
	Vanadium	Total	kg/km^2	2010	2020	-
	Zinc	Total	kg/km^2	2010	2020	-

Supplementary Table 2. All features that were included in the random forest model. Purple represents risk factors, green represents pollution exposure measures, blue represents imaging measures and red represents blood markers. SIMD = Scottish Index of Multiple Deprivation; SO2 = Sulphur dioxide; NO2 = Nitrogen dioxide; NOX = Nitric oxide and nitrogen dioxide ; PM25 = Particulate matter <2.5 micrometres; CO = Carbon monoxide.

**Feature**
Education
Infection
Age
Sex
Height
Weight
Grip Strength
BMI
Total Population Estimate
Working Age Population
Overal SIMD16 Rank
SIMD 2016 Percentile
SIMD 2016 Vigintile
SIMD 2016 Decile
SIMD 2016 Quintile
Income Domain 2016 Rank
Employment Domain 2016 Rank (SIMD)
Health Domain 2016 Rank (SIMD)
Education Domain 2016 Rank (SIMD)
Geographic Access Domain 2016 Rank (SIMD)
Crime Domain 2016 Rank (SIMD)
Housing Domain 2016 Rank (SIMD)
Income Rate (Percentage of income deprived people)
Income Count (Number of income deprived people)
Employment Rate (Percentage of employment deprived people)
Employment Count (Number of employment deprived people)
CIF (Comparative Illness Factor: standardised ratio)
ALCOHOL (Hospital stays related to alcohol use: standardised ratio)
DRUG (Hospital stays related to drug use: standardised ratio)
SMR (Standardised mortality ratio)
DEPRESS (Proportion of population prescribed drugs for anxiety, depression or psychosis)
LBWT (Proportion of live singleton births of low birth weight)
EMERG (Emergency stays in hospital: standardised ratio)
School Attendance
School Attainment
Noquals (Working age people with no qualifications: standardised ratio)
NEET (young people not in education, employment or training)
HESA (Higher Education Statistics Agency - SIMD)
Driving Travel Time To Petrol
Driving Travel Time To GP
Driving Travel Time To Post Office
Driving Travel Time To Primary School
Driving Travel Time To Retail
Driving Travel Time To Secondary School
Public Transport Travel Time to GP
Public Transport Travel Time to Post Office

Public Transport Travel Time to Retail
Crime Rate
Number of People in Overcrowded Households
Number of Houses with Non-central Heating
Percentage of People in Overcrowded Households
Percentage of Houses with Non-central Heating
SO2_2002
SO2_2003
SO2_2004
SO2_2005
SO2_2006
SO2_2007
SO2_2008
SO2_2009
SO2_2010
SO2_2011
SO2_2012
SO2_2013
SO2_2014
SO2_2015
SO2_2016
SO2_2017
SO2_2018
SO2_2019
SO2_2020
SO2_2021
SO2_2022
Benzene_2003
Benzene_2004
Benzene_2005
Benzene_2006
Benzene_2007
Benzene_2008
Benzene_2009
Benzene_2010
Benzene_2011
Benzene_2012
Benzene_2013
Benzene_2014
Benzene_2015
Benzene_2016
Benzene_2017
Benzene_2018
Benzene_2019
Benzene_2020
Benzene_2021
Benzene_2022
NO2_2001
NO2_2002
NO2_2003

NO2_2004
NO2_2005
NO2_2006
NO2_2007
NO2_2008
NO2_2009
NO2_2010
NO2_2011
NO2_2012
NO2_2013
NO2_2014
NO2_2015
NO2_2016
NO2_2017
NO2_2018
NO2_2019
NO2_2020
NO2_2021
NO2_2022
NOX_2001
NOX_2002
NOX_2003
NOX_2004
NOX_2005
NOX_2006
NOX_2007
NOX_2008
NOX_2009
NOX_2010
NOX_2011
NOX_2012
NOX_2013
NOX_2014
NOX_2015
NOX_2016
NOX_2017
NOX_2018
NOX_2019
NOX_2020
NOX_2021
NOX_2022
OZONE_2003
OZONE_2004
OZONE_2005
OZONE_2006
OZONE_2007
OZONE_2008
OZONE_2009
OZONE_2010
OZONE_2011

OZONE_2012
OZONE_2013
OZONE_2014
OZONE_2015
OZONE_2016
OZONE_2017
OZONE_2018
OZONE_2019
OZONE_2020
OZONE_2021
OZONE_2022
PM25_2002
PM25_2003
PM25_2004
PM25_2005
PM25_2006
PM25_2007
PM25_2008
PM25_2009
PM25_2010
PM25_2011
PM25_2012
PM25_2013
PM25_2014
PM25_2015
PM25_2016
PM25_2017
PM25_2018
PM25_2019
PM25_2020
PM25_2021
PM25_2022
CO_2002
CO_2003
CO_2004
CO_2005
CO_2006
CO_2007
CO_2008
CO_2009
CO_2010
CO (MAX) 2002
CO (MAX) 2003
CO (MAX) 2004
CO (MAX) 2005
CO (MAX) 2006
CO (MAX) 2007
CO (MAX) 2008
CO (MAX) 2009
CO (MAX) 2010

Arsenic_2010
Arsenic_2011
Arsenic_2012
Arsenic_2013
Arsenic_2014
Arsenic_2015
Arsenic_2016
Arsenic_2017
Arsenic_2018
Arsenic_2019
Arsenic_2020
Cadmium_2010
Cadmium_2011
Cadmium_2012
Cadmium_2013
Cadmium_2014
Cadmium_2015
Cadmium_2016
Cadmium_2017
Cadmium_2018
Cadmium_2019
Cadmium_2020
Chromium_2010
Chromium_2011
Chromium_2012
Chromium_2013
Chromium_2014
Chromium_2015
Chromium_2016
Chromium_2017
Chromium_2018
Chromium_2019
Chromium_2020
Copper_2010
Copper_2011
Copper_2012
Copper_2013
Copper_2014
Copper_2015
Copper_2016
Copper_2017
Copper_2018
Copper_2019
Copper_2020
Lead_2010
Lead_2011
Lead_2012
Lead_2013
Lead_2014
Lead_2015

Lead_2016
Lead_2017
Lead_2018
Lead_2019
Lead_2020
Mercury_2010
Mercury_2011
Mercury_2012
Mercury_2013
Mercury_2014
Mercury_2015
Mercury_2016
Mercury_2017
Mercury_2018
Mercury_2019
Mercury_2020
Nickel_2010
Nickel_2011
Nickel_2012
Nickel_2013
Nickel_2014
Nickel_2015
Nickel_2016
Nickel_2017
Nickel_2018
Nickel_2019
Nickel_2020
Selenium_2010
Selenium_2011
Selenium_2012
Selenium_2013
Selenium_2014
Selenium_2015
Selenium_2016
Selenium_2017
Selenium_2018
Selenium_2019
Selenium_2020
Vanadium_2010
Vanadium_2011
Vanadium_2012
Vanadium_2013
Vanadium_2014
Vanadium_2015
Vanadium_2016
Vanadium_2017
Vanadium_2018
Vanadium_2019
Vanadium_2020
Zinc_2010
Zinc_2011

Zinc_2012
Zinc_2013
Zinc_2014
Zinc_2015
Zinc_2016
Zinc_2017
Zinc_2018
Zinc_2019
Zinc_2020
Left Thalamus Iron
Left Caudate Iron
Left Putamen Iron
Left Pallidum Iron
Left Hippocampus Iron
Left Amygdala Iron
Right Thalamus Iron
Right Caudate Iron
Right Putamen Iron
Right Pallidum Iron
Right Hippocampus Iron
Right Amygdala Iron
Left Hippocampus Volume
Left Amygdala Volume
Left Thalamus Volume
Left Caudate Volume
Left Putamen Volume
Left Pallidum Volume
Right Thalamus Volume
Right Caudate Volume
Right Putamen Volume
Right Pallidum Volume
Right Hippocampus Volume
Right Amygdala Volume
Left Cerebral White Matter Volume
Left Lateral Ventricle Volume
Left Cerebellum White Matter Volume
Left Cerebellum Cortex Volume
4th Ventricle Volume
Brain Stem Volume
Cerebral Spinal Fluid Volume
Left Accumbens Area Volume
Left Ventral Diencephalon Volume
Right Cerebral White Matter Volume
Right Lateral Ventricle Volume
Right Cerebellum White Matter Volume
Right Cerebellum Cortex Volume
Right Accumbens Area Volume
Right Ventral Diencephalon Volume
White Matter Hypointensities Volume

Corpus Callossum (Posterior) Volume
Corpus Callossum (Mid-Posterior) Volume
Corpus Callossum (Central) Volume
Corpus Callossum (Mid-Anterior) Volume
Corpus Callossum (Anterior) Volume
Left Bankssts Cortex Volume
Left Caudal Anterior Cingulate Cortex Volume
Left Caudal middle frontal Cortex Volume
Left Cuneus Cortex Volume
Left Entorhinal Cortex Volume
Left Fusiform Cortex Volume
Left Inferior Parietal Cortex Volume
Left Inferior Temporal Cortex Volume
Left Isthmus Cingulate Cortex Volume
Left Lateral Occipital Cortex Volume
Left Lateral Orbitofrontal Cortex Volume
Left Lingual Cortex Volume
Left Medialo Orbitofrontal Cortex Volume
Left Middle Temporal Cortex Volume
Left Parahippocampal Cortex Volume
Left Paracentral Cortex Volume
Left Parsopercularis Volume
Left Parsorbitalis Volume
Left Parstriangularis Volume
Left Pericalcarine Cortex Volume
Left Postcentral Cortex Volume
Left Posterior Cingulate Cortex Volume
Left Precentral Cortex Volume
Left Precuneus Cortex Volume
Left Rostral Anterior Cingulate Cortex Volume
Left Rostral Middle Frontal Cortex Volume
Left Superior Frontal Cortex Volume
Left Superior Parietal Cortex Volume
Left Superior Temporal Cortex Volume
Left Supramarginal Cortex Volume
Left Frontalp Pole Volume
Left Temporal Pole Volume
Left Insula Cortex Volume
Right Bankssts Cortex Volume
Right Caudal Anterior Cingulate Cortex Volume
Right Caudal middle frontal Cortex Volume
Right Cuneus Cortex Volume
Right Entorhinal Cortex Volume
Right Fusiform Cortex Volume
Right Inferior Parietal Cortex Volume
Right Inferior Temporal Cortex Volume
Right Isthmus Cingulate Cortex Volume
Right Lateral Occipital Cortex Volume
Right Lateral Orbitofrontal Cortex Volume
Right Lingual Cortex Volume
Right Medialo Orbitofrontal Cortex Volume

Right Middle Temporal Cortex Volume
Right Parahippocampal Cortex Volume
Right Paracentral Cortex Volume
Right Parsopercularis Volume
Right Parsorbitalis Volume
Right Parstriangularis Volume
Right Pericalcarine Cortex Volume
Right Postcentral Cortex Volume
Right Posterior Cingulate Cortex Volume
Right Precentral Cortex Volume
Right Precuneus Cortex Volume
Right Rostral Anterior Cingulate Cortex Volume
Right Rostral Middle Frontal Cortex Volume
Right Superior Frontal Cortex Volume
Right Superior Parietal Cortex Volume
Right Superior Temporal Cortex Volume
Right Supramarginal Cortex Volume
Right Frontalp Pole Volume
Right Temporal Pole Volume
Right Insula Cortex Volume
3rd Ventricle Volume
Left Choroid Plexus Volume
Right Choroid Plexus Volume
Right Transverse Temporal Cortex Volume
Left Transverse Temporal Cortex Volume
Plasma Ferritin
Plasma Soluble Transferrin Receptor
Plasma Macrophage Colony Stimulating Factor
Plasma IL6
Plasma IL1B
C-reactive Protein
Haemoglobin
Red Blood Cell Count
Haematocrit
Mean Corpuscular Volume
Mean Corpuscular Haemoglobin
Platelet Count
White Blood Cell Count
Neutrophil Count
Eosinophil Count
Basophil Count
Lymphocyte Count
Monocyte Count
Large Unstained Cell Count
Mean Corpuscular Haemoglobin Concentration
Plasma Calcium (44 Ca [No Gas])
Plasma Copper (63 Cu [He])
Plasma Iron (56 Fe [He])
Plasma Magnesium (24 Mg [He])
Plasma Phosphorus (31 P [No Gas])
Plasma Potassium (39 K [No Gas])
Plasma Selenium (78 Se [H2])

Plasma Zinc (66 Zn [He])
Plasma Lead (208 Pb [No Gas])
Plasma Sodium (Na)
Plasma Manganese (Mn)
Plasma Cobalt (Co)
Plasma Molybdenum (Mo)
Plasma Cadmium (Cd)
Plasma Mercury (Hg)
Plasma Arsenic (As)

Supplementary Table 3. Random forest leave-one-out feature analysis for g score showing the % improvement when a feature is included in the model. This is defined by the % increase in proportion of correctly classified cases when the feature is included in the model compared to when left out. Features that worsened predictivity of the model when left out were considered predictors of cognitive performance. Purple represents risk factors, green represents pollution exposure measures, blue represents imaging measures and red represents blood markers.

Feature	% Improvement
Age	3.08
School Attainment	3.08
Sex	3.08
Number of Housese with Non-central Heating	3.08
Arsenic (2012)	3.08
Arsenic (2013)	3.08
Arsenic (2014)	3.08
Arsenic (2015)	3.08
Arsenic (2020)	3.08
Carbon Monoxide (MAX; 2006)	3.08
Carbon Monoxide (MAX; 2007)	3.08
Carbon Monoxide (MAX; 2008)	3.08
Cadmium (2011)	3.08
Cadmium (2012)	3.08
Cadmium (2014)	3.08
Cadmium (2016)	3.08
Copper (2014)	3.08
Copper (2016)	3.08
Copper (2017)	3.08
Copper (2018)	3.08
Copper (2019)	3.08
Lead (2010)	3.08
Lead (2011)	3.08
Lead (2013)	3.08
Mercury (2011)	3.08
Mercury (2012)	3.08
Mercury (2017)	3.08
Mercury (2018)	3.08
Particulate Matter (PM2.5; 2005)	3.08
Particulate Matter (PM2.5; 2009)	3.08
Particulate Matter (PM2.5; 2017)	3.08
Particulate Matter (PM2.5; 2019)	3.08
Particulate Matter (PM2.5; 2022)	3.08
Sulfur Dioxide (2003)	3.08
Sulfur Dioxide (2010)	3.08
Corpus Callossum Central volume	3.08
Corpus Callossum Mid-Posterior volume	3.08

Left Pallidum Iron	3.08
Left Putamen Iron	3.08
Left Accumbens Area Volume	3.08
Left Amygdala Volume	3.08
Left Hippocampus Volume	3.08
Left Thalamus Volume	3.08
4th Ventricle Volume	3.08
Left Caudalanteriorcingulate Cortex Volume	3.08
Left Cuneus Volume	3.08
Left Inferiortemporal Cortex Volume	3.08
Left Lingual Cortex Volume	3.08
Left Paracentral Cortex Volume	3.08
Left Parsopercularis Volume	3.08
Right Entorhinal Cortex Volume	3.08
C-Reactive Protein	3.08
White Blood Cell Count	3.08
Education	6.08
Weight (kg)	6.08
Rate of Overcrowding	6.08
Carbon Monoxide (2002)	6.08
Carbon Monoxide (2003)	6.08
Carbon Monoxide (2004)	6.08
Carbon Monoxide (2005)	6.08
Carbon Monoxide (2006)	6.08
Carbon Monoxide (2007)	6.08
Carbon Monoxide (MAX; 2009)	6.08
Carbon Monoxide (MAX; 2010)	6.08
Cadmium (2013)	6.08
Copper (2015)	6.08
Lead (2012)	6.08
Lead (2012)	6.08
Lead (2015)	6.08
Lead (2017)	6.08
Lead (2018)	6.08
Lead (2019)	6.08
Lead (2020)	6.08
Particulate Matter (PM2.5; 2007)	6.08
Particulate Matter (PM2.5; 2011)	6.08
Particulate Matter (PM2.5; 2018)	6.08
Corpus Callossum Anterior Volume	6.08
Corpus Callossum Mid-Anterior Volume	6.08
Corpus Callossum Posterior Volume	6.08
Left Ventral Diencephalon Volume	6.08
Right Amygdala Iron	6.08
Right Accumbens Area Volume	6.08
Right Cerebellum Cortex Volume	6.08
Right Cerebellum White Matter Volume	6.08
Right Pallidum Volume	6.08

Left Bankssts Cortex Volume	6.08
Left Caudal Middle Frontal Cortex Volume	6.08
Pericalcarine Cortex Volume	6.08
Left Postcentral Cortex Volume	6.08
Haematocrit	6.08
Haemoglobin	6.08
Mean Corpuscular Haemoglobin	6.08
Arsenic (2010)	9.08
Arsenic (2011)	9.08
Carbon Monoxide (2008)	9.08
Carbon Monoxide (2009)	9.08
Carbon Monoxide (2010)	9.08
Carbon Monoxide (MAX; 2002)	9.08
Carbon Monoxide (MAX; 2003)	9.08
Carbon Monoxide (MAX; 2004)	9.08
Carbon Monoxide (MAX; 2005)	9.08
Lead (2016)	9.08
Particulate Matter (PM2.5; 2010)	9.08
Particulate Matter (PM2.5; 2012)	9.08
Mean Corpuscular Volume	12.08

Supplementary Table 4. Random forest leave-one-out feature analysis for memory score (immediate recall) showing the % improvement when a feature is included in the model. This is defined by the % increase in proportion of correctly classified cases when the feature is included in the model compared to when left out. Features that worsened predictivity of the model when left out were considered predictors of cognitive performance. Purple represents risk factors, green represents pollution exposure measures, blue represents imaging measures and red represents blood markers. SIMD = Scottish Index of Multiple Deprivation; SO2 = Sulphur dioxide; NO2 = Nitrogen dioxide; NOX = Nitric oxide and nitrogen dioxide ; PM25 = Particulate matter <2.5 micrometres; CO = Carbon monoxide.

Feature	% Improvement
Crime Domain 2016 Rank (SIMD)	1.96
EMERG (Emergency stays in hospital: standardised ratio)	1.96
Education Domain 2016 Rank (SIMD)	1.96
Geographic Domain 2016 Rank (SIMD)	1.96
Health Domain 2016 Rank (SIMD)	1.96
Income Domain 2016 Rank (SIMD)	1.96
LBWT (Proportion of live singleton births of low birth weight)	1.96
Overal SIMD16 Rank	1.96
SIMD 2016 Vigintile	1.96
SMR (Standardised mortality ratio)	1.96
Driving Travel Time To GP	1.96
Driving Travel Time To Post Office	1.96
Driving Travel Time To Primary School	1.96
Driving Travel Time To Retail	1.96
Number of People in Overcrowded Households	1.96
Arsenic (2011)	1.96
Arsenic (2012)	1.96
Arsenic (2016)	1.96
Arsenic (2017)	1.96
Arsenic (2018)	1.96
Arsenic (2019)	1.96
Arsenic (2020)	1.96
Benzene (2009)	1.96
Benzene (2014)	1.96
Benzene (2015)	1.96
Benzene (2016)	1.96
Benzene (2018)	1.96
Benzene (2020)	1.96
Benzene (2021)	1.96
Cadmium (2014)	1.96
Cadmium (2016)	1.96
Copper (2011)	1.96
Copper (2012)	1.96
Copper (2013)	1.96
Copper (2014)	1.96
NO2 (2017)	1.96
NOX (2002)	1.96
NOX (2003)	1.96
NOX (2005)	1.96
NOX (2009)	1.96
NOX (2010)	1.96
NOX (2011)	1.96
NOX (2012)	1.96
NOX (2013)	1.96

Nickel (2012)	1.96
Nickel (2013)	1.96
Nickel (2014)	1.96
Nickel (2016)	1.96
Nickel (2017)	1.96
Nickel (2018)	1.96
Nickel (2019)	1.96
Nickel (2020)	1.96
OZONE (2008)	1.96
OZONE (2016)	1.96
PM25 (2005)	1.96
PM25 (2014)	1.96
PM25 (2015)	1.96
PM25 (2017)	1.96
SO2 (2016)	1.96
Selenium (2010)	1.96
Selenium (2015)	1.96
Selenium (2016)	1.96
Selenium (2017)	1.96
Selenium (2018)	1.96
Selenium (2020)	1.96
Vanadium (2010)	1.96
Vanadium (2011)	1.96
Vanadium (2012)	1.96
Vanadium (2013)	1.96
Vanadium (2015)	1.96
Vanadium (2016)	1.96
Vanadium (2017)	1.96
Vanadium (2018)	1.96
Vanadium (2019)	1.96
Vanadium (2020)	1.96
Zinc (2010)	1.96
Zinc (2014)	1.96
Zinc (2015)	1.96
Zinc (2017)	1.96
Corpus Callossum (Central) Volume	1.96
Corpus Callossum (Mid-anterior) Volume	1.96
Corpus Callossum (Mid-posterior) Volume	1.96
Corpus Callossum (Posterior) Volume	1.96
Left Caudate Iron	1.96
Left Putamen Iron	1.96
Left Thalamus Iron	1.96
Left Caudate Volume	1.96
Left Hippocampus Volume	1.96
Right Amygdala Iron	1.96
Right Pallidum Iron	1.96
Right Cerebellum Cortex Volume	1.96
White Matter Hypointensities Volume	1.96
Left Paracentral Cortex Volume	1.96

Left Parsorbitalis Volume	1.96
Right Insula Cortex Volume	1.96
Right Paracentral Cortex Volume	1.96
Right Pericalcarine volume	1.96
C-reactive Protein	1.96
Lymphocyte Count	1.96
Percentage of Houses with Non-central Heating	3.92
Percentage of People in Overcrowded Households	3.92
School Pupil Attendance	3.92
Number of Employment Deprived People	3.92
HESA (Higher Education Statistics Agency - SIMD)	3.92
Housing Domain 2016 Rank	3.92
Public Transport Travel Time To GP	3.92
SIMD 2016 Percentile	3.92
Crime Rate	3.92
Benzene (2004)	3.92
Benzene (2005)	3.92
Benzene (2006)	3.92
Benzene (2007)	3.92
Benzene (2008)	3.92
Benzene (2010)	3.92
Benzene (2011)	3.92
Benzene (2012)	3.92
Cadmium (2013)	3.92
Mercury (2017)	3.92
Mercury (2018)	3.92
Mercury (2019)	3.92
Mercury (2020)	3.92
NO2 (2001)	3.92
NO2 (2018)	3.92
NO2 (2019)	3.92
NO2 (2020)	3.92
NOX (2006)	3.92
NOX (2007)	3.92
NOX (2008)	3.92
NOX (2014)	3.92
NOX (2015)	3.92
Nickel (2011)	3.92
OZONE (2007)	3.92
OZONE (2011)	3.92
OZONE (2015)	3.92
SO2 (2017)	3.92
SO2 (2018)	3.92
SO2 (2022)	3.92
Vanadium (2014)	3.92
Zinc (2011)	3.92
Zinc (2012)	3.92
Zinc (2013)	3.92

Zinc (2016)	3.92
Zinc (2018)	3.92
Zinc (2019)	3.92
Zinc (2020)	3.92
Cerebral Spinal Fluid Volume	3.92
Left Accumbens Area Volume	3.92
Left Amygdala Volume	3.92
Left Cerebellum White Matter Volume	3.92
Left Inferior Lateral Ventricle Volume	3.92
Right Accumbens Area Volume	3.92
Right Cerebellum White Matter Volume	3.92
Right Cerebral White Matter Volume	3.92
Right Ventral Diencephalon Volume	3.92
Left Medial Orbitofrontal Cortex Volume	3.92
Left Parsopercularis Volume	3.92
Left Precuneus Cortex Volume	3.92
Left Rostral Anterior Cingulate Cortex Volume	3.92
Right Frontal Pole Volume	3.92
Right Lingual Cortex Volume	3.92
Right Parsorbitalis Volume	3.92
Right Postcentral Cortex Volume	3.92
Right Precentral Cortex Volume	3.92
Right Superior Frontal Cortex Volume	3.92
Right Superior Parietal Cortex Volume	3.92
Right Temporal Pole Volume	3.92
Plasma Lead (208 Pb [No Gas])	3.92
Haemoglobin	3.92
Council Area (SIMD)	5.88
DEPRESS (Proportion of population prescribed drugs for anxiety, depression or psychosis)	5.88
Employment Rate (Percentage of employment deprived people)	5.88
Income Rate (Percentage of income deprived people)	5.88
Public Transport Travel Time To Retail	5.88
Total Population Estimation (SIMD)	5.88
Driving Travel Time To Secondary School	5.88
Number of People in Houses with Non-central Heating	5.88
Benzene (2003)	5.88
Cadmium (2011)	5.88
Mercury (2016)	5.88
NO2 (2006)	5.88
NO2 (2008)	5.88
NO2 (2009)	5.88
NO2 (2010)	5.88
NO2 (2011)	5.88
NO2 (2012)	5.88
NO2 (2013)	5.88
NO2 (2014)	5.88
NO2 (2015)	5.88
NO2 (2016)	5.88

NOX (2004)	5.88
NOX (2017)	5.88
Nickel (2010)	5.88
OZONE (2006)	5.88
SO2 (2019)	5.88
SO2 (2021)	5.88
Left Amygdala Iron	5.88
Left Putamen Volume	5.88
Left Ventral Diencephalon Volume	5.88
Right Thalamus Iron	5.88
Right Amygdala Volume	5.88
Right Caudate Volume	5.88
Right Lateral Ventricle Volume	5.88
Right Pallidum Volume	5.88
Right Putamen Volume	5.88
4th Ventricle Volume	5.88
Left Parstriangularis Volume	5.88
Left Precentral Cortex Volume	5.88
Left Rostral Middle Frontal Cortex Volume	5.88
Left Superior Parietal Cortex Volume	5.88
Right Lateral Orbitofrontal Cortex Volume	5.88
Right Medial Orbitofrontal Cortex Volume	5.88
Right Middle Temporal Cortex Volume	5.88
Right Parahippocampal Cortex Volume	5.88
Right Parsopercularis Volume	5.88
Right Parstriangularis Volume	5.88
Right Posterior Cingulate Cortex Volume	5.88
Right Rostral Anterior Cingulate Cortex Volume	5.88
Right Rostral Middle Frontal Cortex Volume	5.88
Right Supramarginal Cortex Volume	5.88
Public Transport Travel Time To Post Office	7.84
DRUG (Hospital stays related to drug use: standardised ratio)	7.84
NEET (young people not in education, employment or training)	7.84
Noquals (Working age people with no qualifications: standardised ratio)	7.84
ALCOHOL (Hospital stays related to alcohol use: standardised ratio)	7.84
CIF (Comparative Illness Factor: standardised ratio)	7.84
Cadmium (2012)	7.84
NO2 (2007)	7.84
NOX (2016)	7.84
NOX (2018)	7.84
NOX (2019)	7.84
OZONE (2004)	7.84
SO2 (2020)	7.84
Left Hippocampus Iron	7.84
Left Pallidum Iron	7.84
Left Cerebral White Matter Volume	7.84
Left Lateral Ventricle Volume	7.84
Left Pallidum Volume	7.84
Right Thalamus Volume	7.84

Left Pericalcarine Cortex Volume	7.84
Left Posterior Cingulate Cortex Volume	7.84
Left Supramarginal Cortex Volume	7.84
Right Precuneus Cortex Volume	7.84
Right Superior Temporal Cortex Volume	7.84
Number of Income Deprived People	9.8
NOX (2020)	9.8
NOX (2021)	9.8
NOX (2022)	9.8
OZONE (2003)	9.8
Brain Stem Volume	9.8
Right Hippocampus Volume	9.8
Left Postcentral Cortex Volume	9.8
Left Superior Frontal Cortex Volume	9.8
Right Lateral Occipital Cortex Volume	9.8
Data Zone (SIMD)	11.76
Grip Strength	11.76
Intermediate Zone (SIMD)	11.76
Left Frontal Pole Volume	11.76
Right Caudal Middle Frontal Cortex Volume	11.76
Right Entorhinal Cortex Volume	11.76
Right Inferior Parietal Cortex Volume	11.76
Right Inferior Temporal Cortex Volume	11.76
Plasma Soluble Transferrin Receptor	11.76
Plasma IL6	11.76
Plasma Macrophage Colony Stimulating Factor	11.76
Platelet Count	11.76
Plasma Calcium (44 Ca [No Gas])	11.76
Plasma Copper (63 Cu [He])	11.76
Plasma Iron (56 Fe [He])	11.76
Plasma Magnesium (24 Mg [He])	11.76
Plasma Phosphorus (31 P [No Gas])	11.76
Plasma Potassium (39 K [No Gas])	11.76
Plasma Selenium (78 Se [H2])	11.76
Plasma Zinc (66 Zn [He])	11.76
Plasma Ferritin	11.76
Mean Corpuscular Haemoglobin Concentration	11.76
White Blood Cell Count	11.76
Left Superior Temporal Cortex Volume	15.69
Left Temporal Pole Volume	15.69
Right Fusiform Volume	15.69
Right Isthmus Cingulate Cortex Volume	15.69
Left Insula Cortex Volume	17.65
Right Bankssts Volume	17.65
Right Caudal Anterior Cingulate Cortex Volume	17.65

Supplementary Table 5. Random forest leave-one-out feature analysis for memory score (delayed recall) showing the % improvement when a feature is included in the model. This is defined by the % increase in proportion of correctly classified cases when the feature is included in the model compared to when left out. Features that worsened predictivity of the model when left out were considered predictors of cognitive performance. Purple represents risk factors, green represents pollution exposure measures, blue represents imaging measures and red represents blood markers. SIMD = Scottish Index of Multiple Deprivation; SO2 = Sulphur dioxide; NO2 = Nitrogen dioxide; NOX = Nitric oxide and nitrogen dioxide ; PM25 = Particulate matter <2.5 micrometres; CO = Carbon monoxide.

Feature	% Improvement
Driving Travel Time To GP	2.00
Driving Travel Time To Secondary School	2.00
Income Domain 2016 Rank (SIMD)	2.00
Income Count (Number of income deprived people)	2.00
Overal SIMD16 Rank	2.00
SIMD 2016 Decile	2.00
SIMD 2016 Percentile	2.00
SIMD 2016 Vigintile	2.00
Arsenic (2012)	2.00
Arsenic (2016)	2.00
Arsenic (2017)	2.00
Arsenic (2018)	2.00
Arsenic (2019)	2.00
Arsenic (2020)	2.00
CO (2007)	2.00
CO MAX (2006)	2.00
Cadmium (2010)	2.00
Cadmium (2011)	2.00
Cadmium (2018)	2.00
Chromium (2010)	2.00
Lead (2015)	2.00
Lead (2016)	2.00
Lead (2017)	2.00
Lead (2018)	2.00
Lead (2019)	2.00
Mercury (2012)	2.00
Mercury (2016)	2.00
Mercury (2018)	2.00
Mercury (2020)	2.00
NO2 (2005)	2.00
NO2 (2008)	2.00
NO2 (2010)	2.00
NO2 (2011)	2.00
NO2 (2012)	2.00
NO2 (2013)	2.00
NOX (2021)	2.00
Nickel (2013)	2.00
Nickel (2019)	2.00
Nickel (2020)	2.00
OZONE (2007)	2.00
PM25 (2002)	2.00
PM25 (2003)	2.00
PM25 (2015)	2.00

SO2 (2006)	2.00
SO2 (2012)	2.00
Selenium (2011)	2.00
Selenium (2012)	2.00
Selenium (2013)	2.00
Selenium (2016)	2.00
Selenium (2019)	2.00
Selenium (2020)	2.00
Vanadium (2018)	2.00
Zinc (2012)	2.00
Zinc (2013)	2.00
Corpus Callossum (Posterior) Volume	2.00
Left Hippocampus Iron	2.00
Left Pallidum Iron	2.00
Left Amygdala Volume	2.00
Left Cerebellum Cortex Volume	2.00
Left Thalamus Volume	2.00
Right Amygdala Iron	2.00
Right Putamen Iron	2.00
Right Cerebellum Cortex Volume	2.00
Basophil Count	2.00
Driving Travel Time To Post Office	4.00
Driving Travel Time To Primary School	4.00
Housing Domain 2016 Rank (SIMD)	4.00
Income Rate (Percentage of income deprived people)	4.00
SIMD 2016 Quintile	4.00
Arsenic (2014)	4.00
Arsenic (2015)	4.00
CO (2006)	4.00
CO (2008)	4.00
Cadmium (2013)	4.00
Cadmium (2015)	4.00
Cadmium (2016)	4.00
Cadmium (2017)	4.00
Cadmium (2020)	4.00
Chromium (2011)	4.00
Mercury (2013)	4.00
Mercury (2015)	4.00
Mercury (2017)	4.00
Mercury (2019)	4.00
NO2 (2001)	4.00
NO2 (2002)	4.00
NO2 (2003)	4.00
NO2 (2004)	4.00
NO2 (2006)	4.00
Nickel (2010)	4.00
Nickel (2011)	4.00
Nickel (2012)	4.00
SO2 (2005)	4.00

Selenium (2010)	4.00
Selenium (2015)	4.00
Selenium (2018)	4.00
Vanadium (2014)	4.00
Vanadium (2015)	4.00
Vanadium (2016)	4.00
Vanadium (2017)	4.00
Vanadium (2019)	4.00
Vanadium (2020)	4.00
Zinc (2010)	4.00
Zinc (2011)	4.00
Zinc (2014)	4.00
Zinc (2015)	4.00
Zinc (2016)	4.00
Zinc (2017)	4.00
Zinc (2019)	4.00
Left Accumbens Area Volume	4.00
Left Pallidum Volume	4.00
Right Caudate Iron	4.00
C-Reactive Protein	4.00
Crime Domain 2016 Rank (SIMD)	6.00
Education Domain 2016 Rank (SIMD)	6.00
Employment Domain 2016 Rank (SIMD)	6.00
Employment Count (Number of employment deprived people)	6.00
Geographic Access Domain 2016 Rank (SIMD)	6.00
Health Domain 2016 Rank (SIMD)	6.00
Arsenic (2013)	6.00
CO (2010)	6.00
CO MAX (2007)	6.00
Cadmium (2014)	6.00
Cadmium (2019)	6.00
Chromium (2014)	6.00
Chromium (2016)	6.00
Chromium (2019)	6.00
Lead (2010)	6.00
Lead (2011)	6.00
Lead (2012)	6.00
Lead (2013)	6.00
NO2 (2007)	6.00
Zinc (2018)	6.00
Zinc (2020)	6.00
Left Ventral Diencephalon Volume	6.00
Right Cerebral White Matter Volume	6.00
Mean Corpuscular Haemoglobin	6.00
Total Population Estimation (SIMD)	8.00
Working Age Population	8.00
Driving Travel Time To Retail	8.00
Council Area (SIMD)	8.00
Employment Rate (Percentage of employment deprived people)	8.00

Benzene (2021)	8.00
Benzene (2022)	8.00
Chromium (2015)	8.00
Chromium (2017)	8.00
Chromium (2018)	8.00
Chromium (2020)	8.00
Copper (2011)	8.00
Copper (2012)	8.00
Copper (2019)	8.00
Selenium (2014)	8.00
Left Amygdala Iron	8.00
Right Thalamus Iron	8.00
Chromium (2012)	10.00
Chromium (2013)	10.00
Copper (2010)	10.00
Copper (2013)	10.00
Copper (2014)	10.00
Copper (2015)	10.00
Copper (2018)	10.00
Copper (2020)	10.00
Copper (2016)	12.00
Copper (2017)	14.00

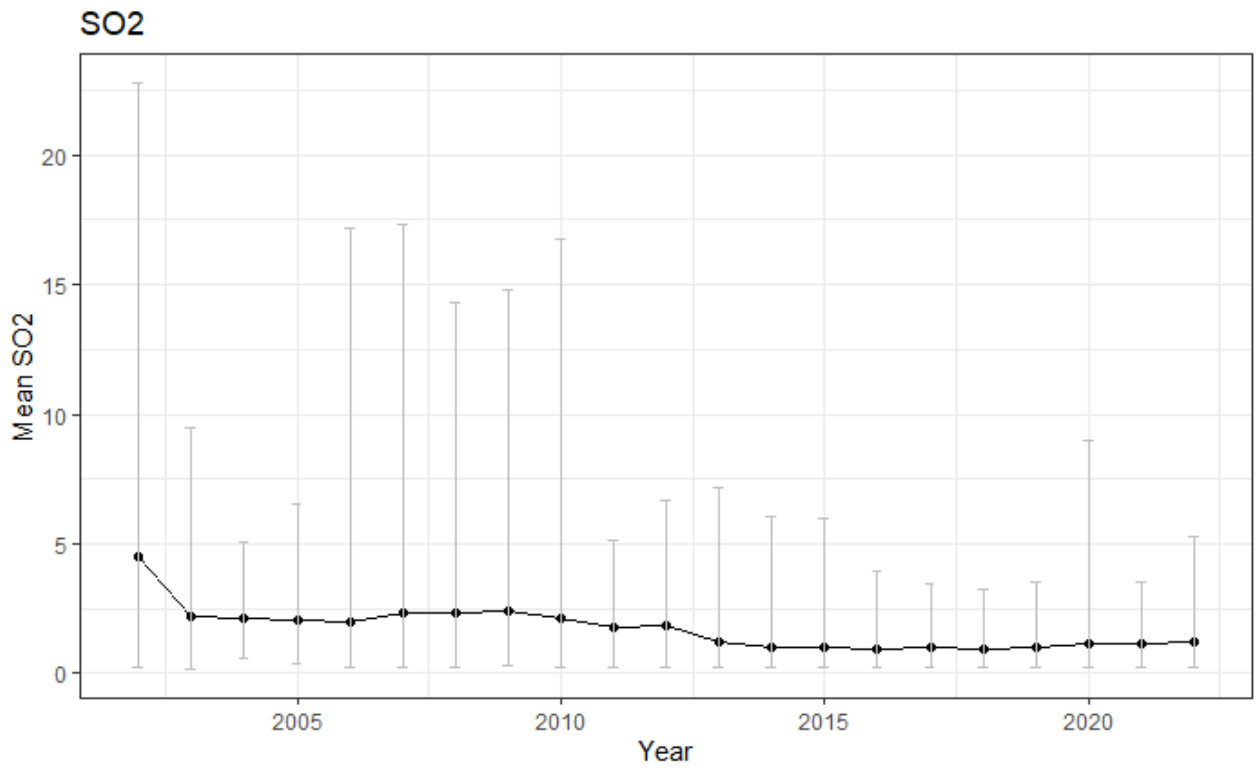

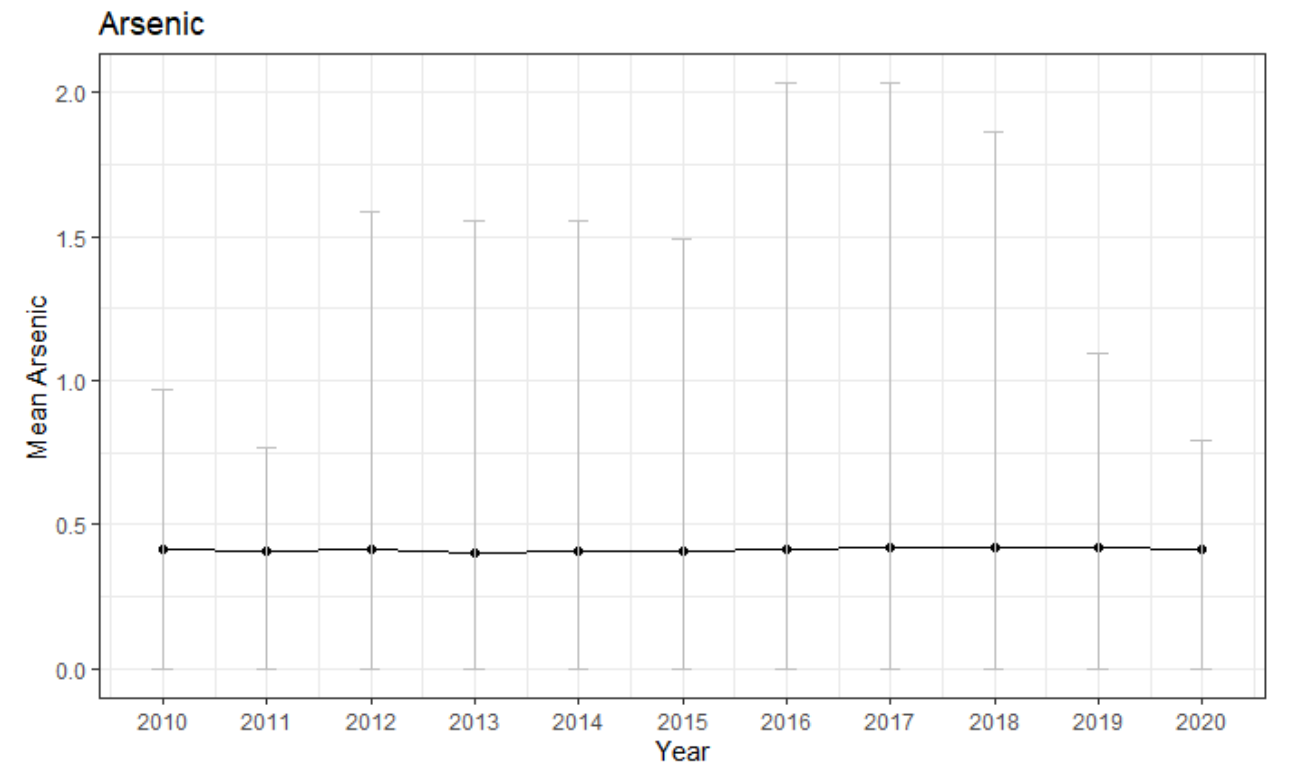

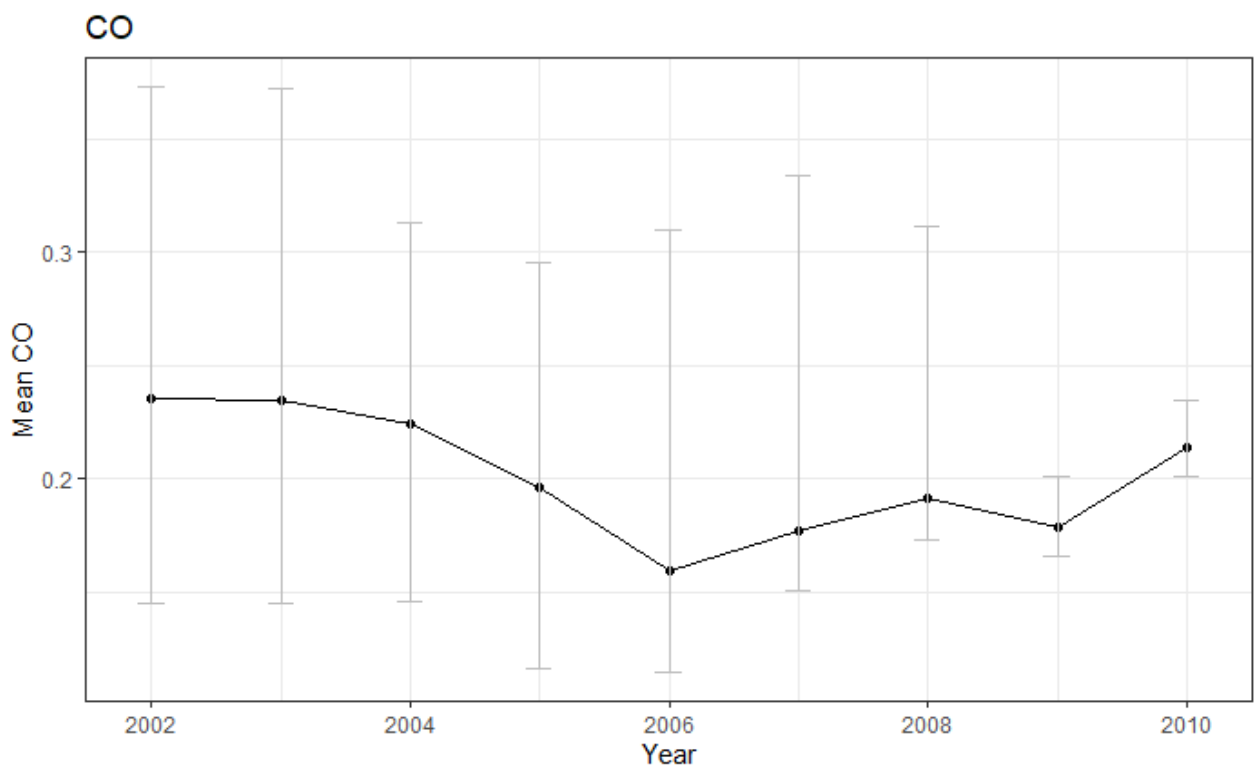

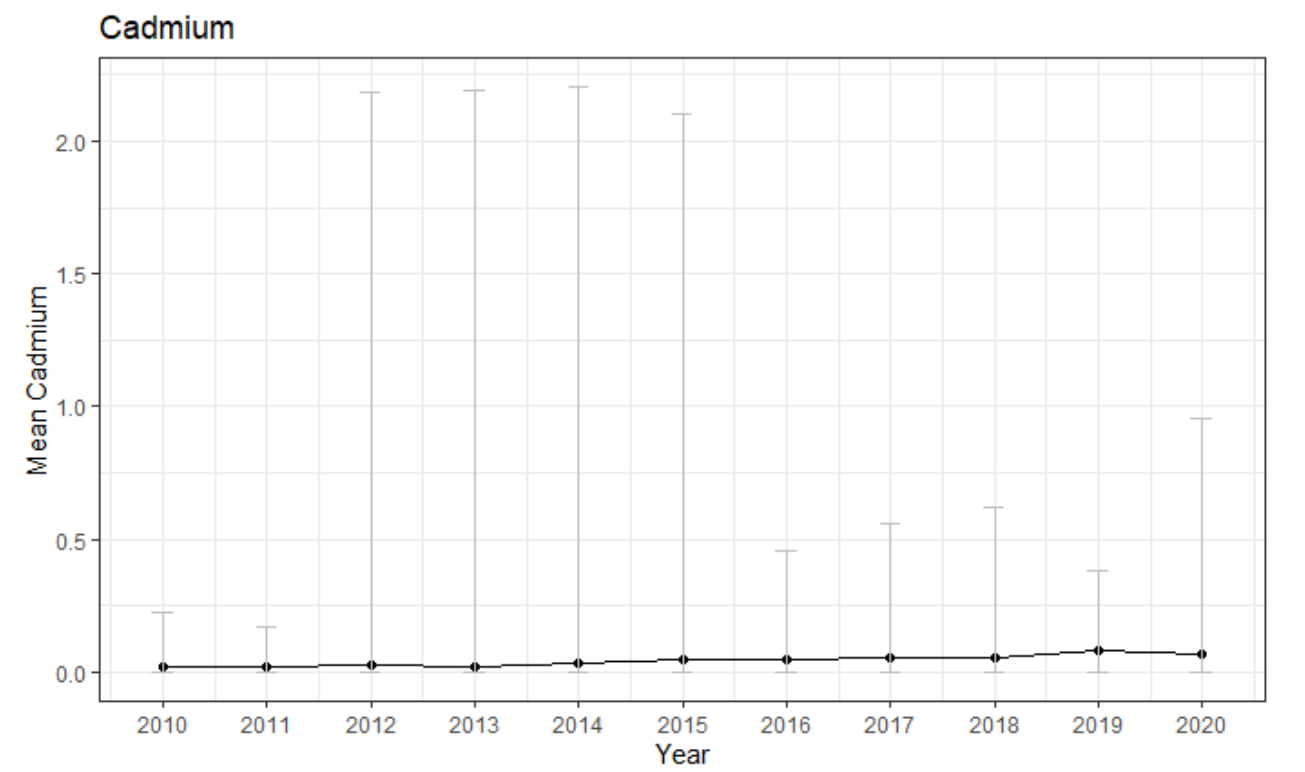

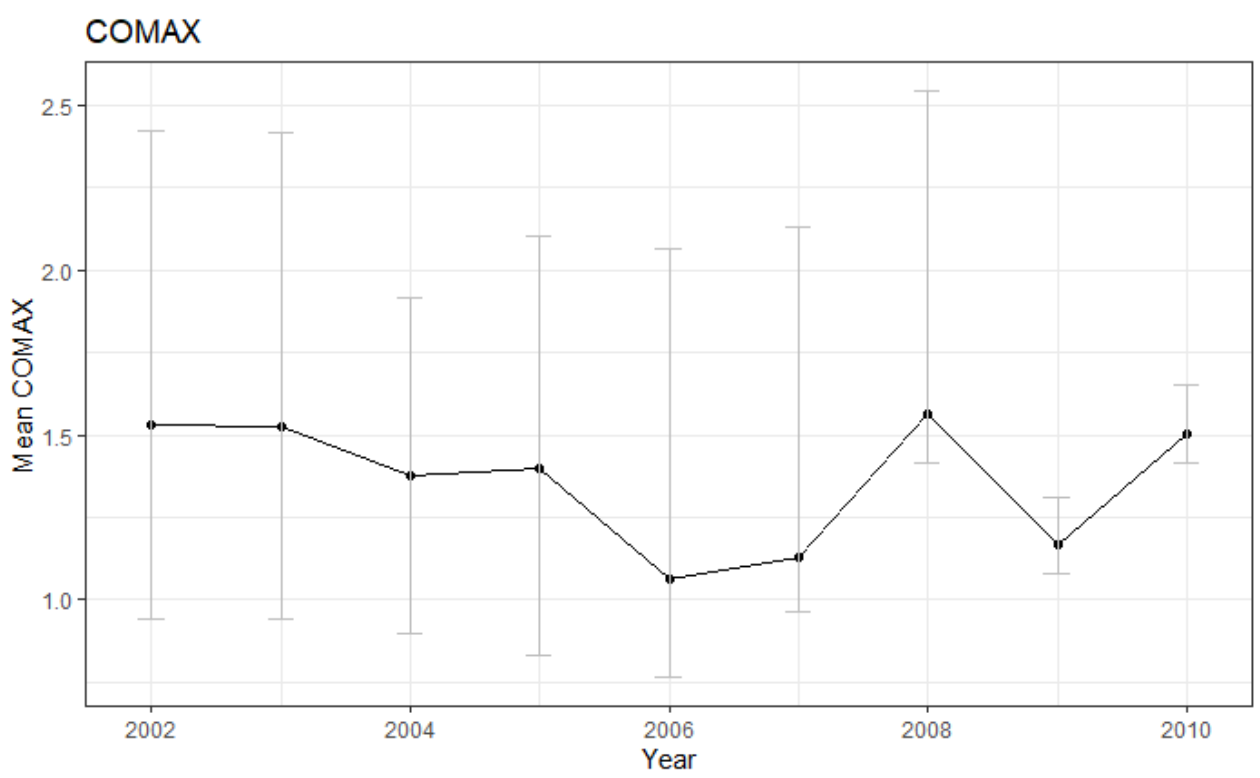

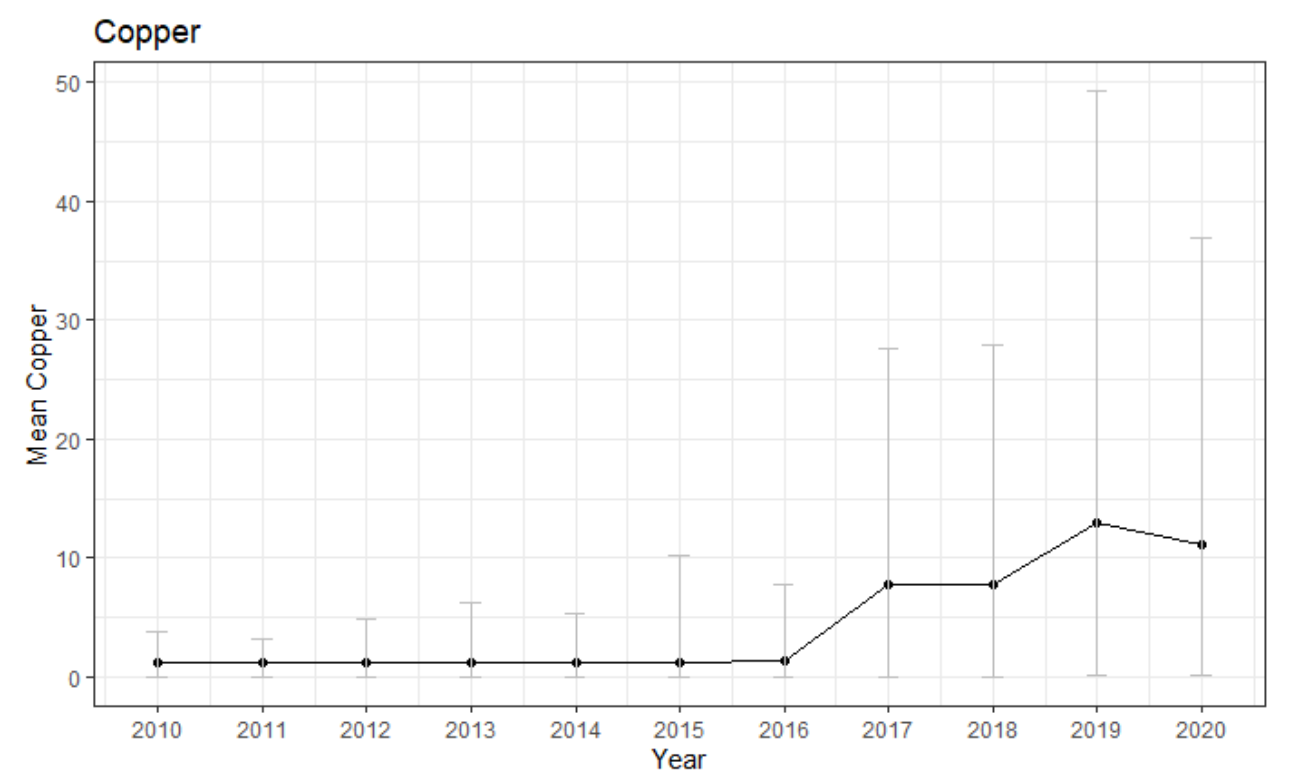

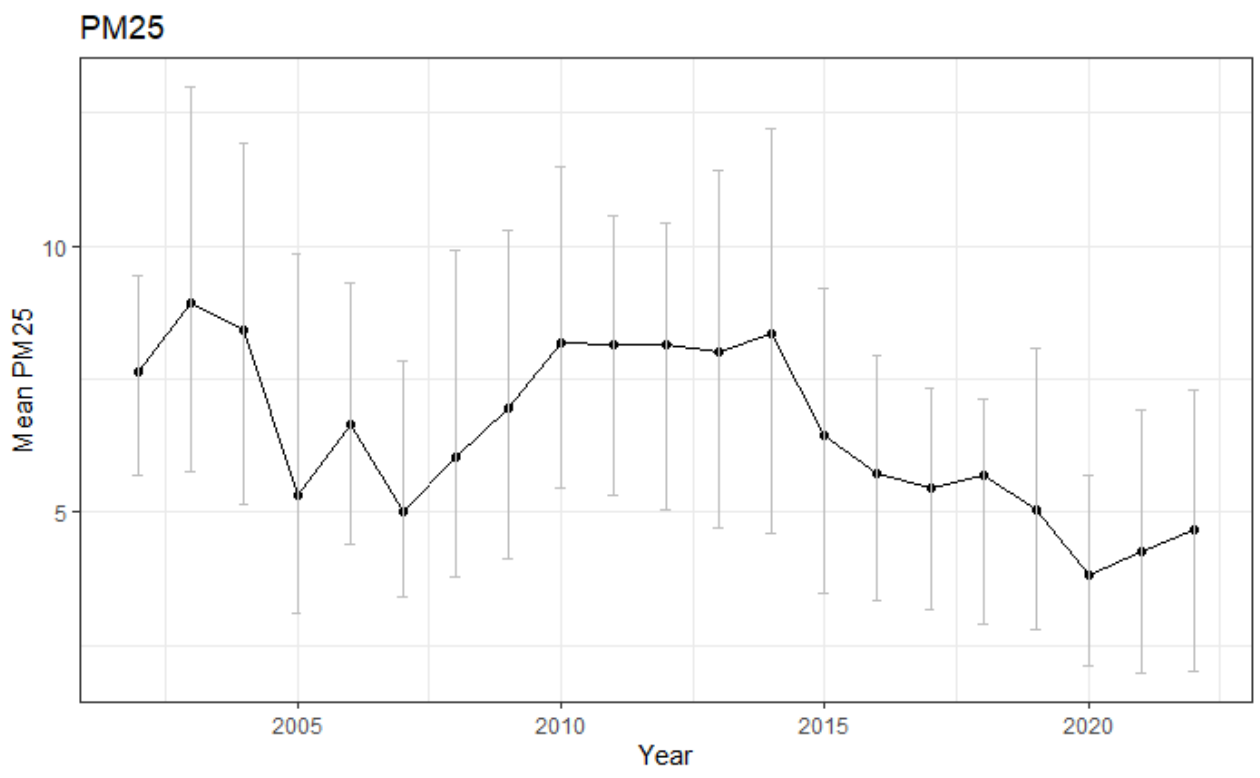

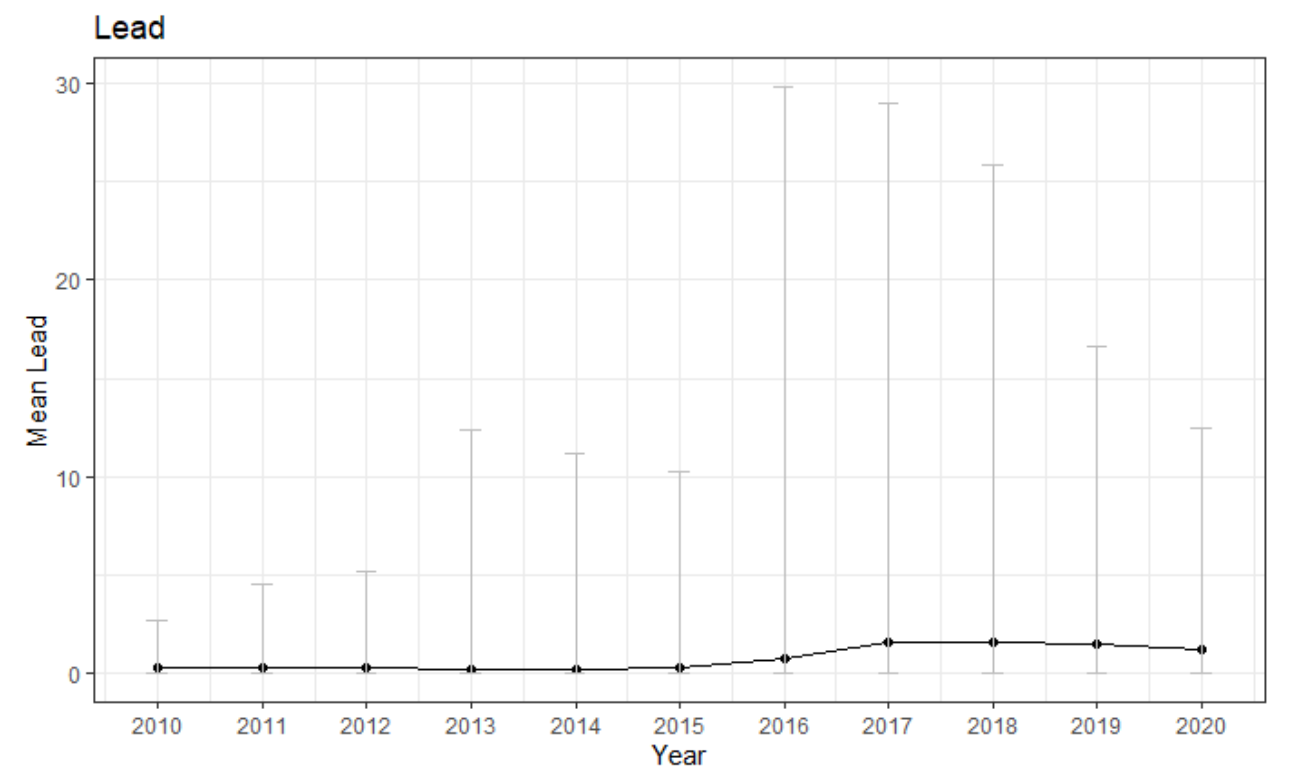

Supplementary Figure 1. Yearly mean concentrations of ambient pollution measures. SO2 = Sulphur dioxide; NO2 = Nitrogen dioxide; NOX = Nitric oxide and nitrogen dioxide ; PM25 = Particulate matter <2.5 micrometres; CO = Carbon monoxide. Bars show the minimum and maximum average annual concentration of each ambient pollutant.

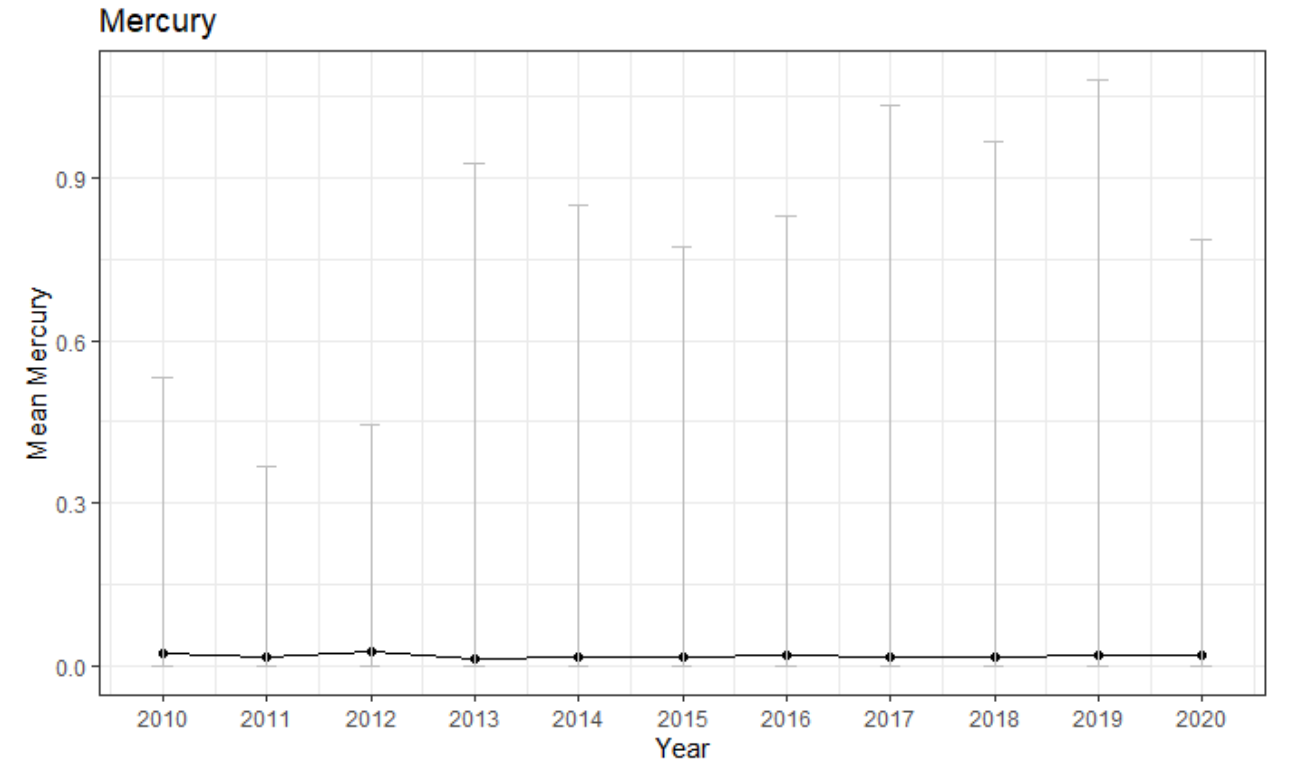

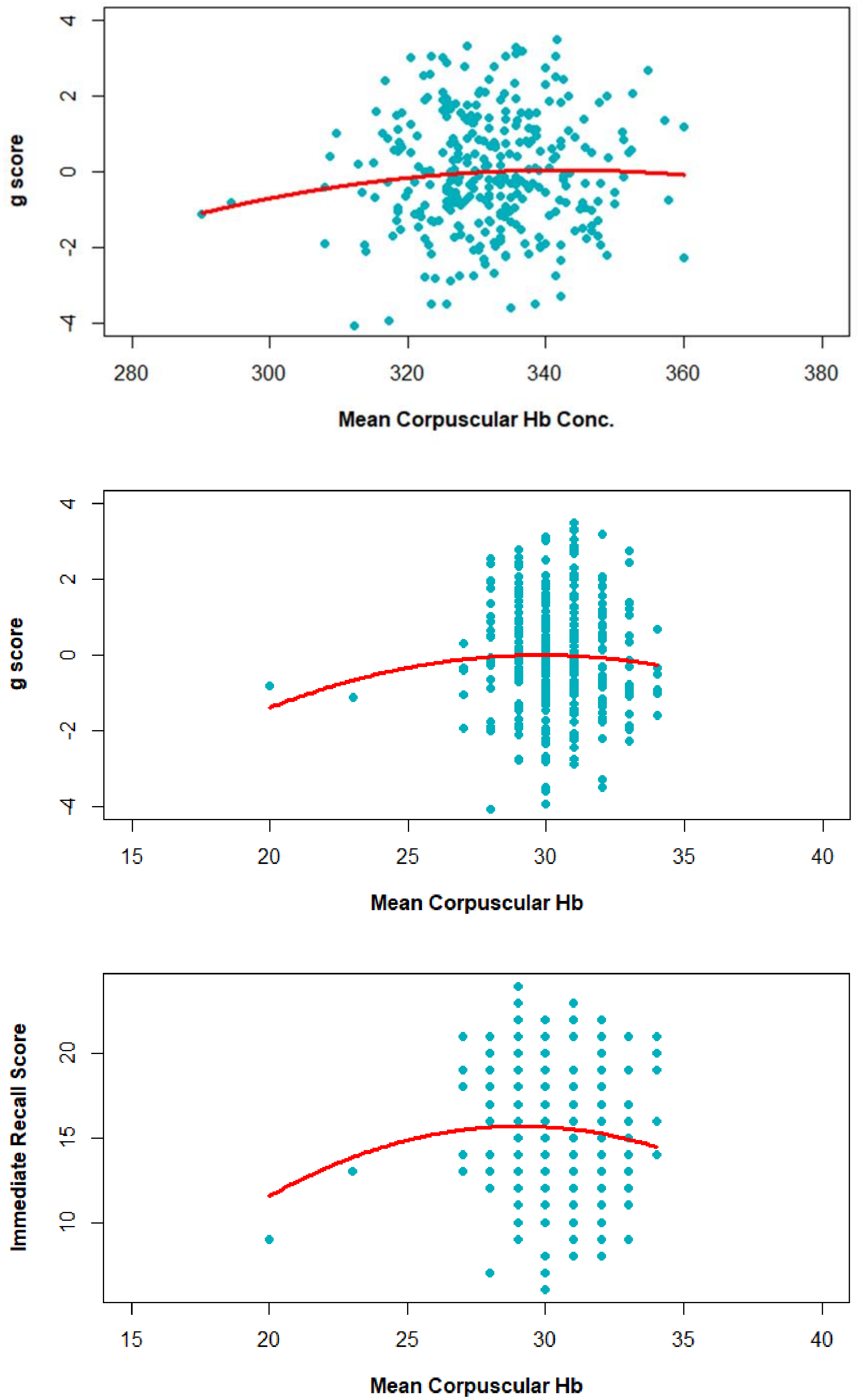

Supplementary Figure 2. a) Mean corpuscular haemoglobin concentration was not significantly associated with general cognition score ($p=0.303$). **b)** Mean corpuscular haemoglobin was not significantly associated with general cognition score ($p=0.5352$). **c)** Mean corpuscular haemoglobin was not significantly associated with immediate recall score ($p=0.213$).
